# Association of statin use with outcomes of patients admitted with COVID-19: an analysis of electronic health records using superlearner

**DOI:** 10.1186/s12879-023-08026-0

**Published:** 2023-02-24

**Authors:** Adovich S. Rivera, Omar Al-Heeti, Lucia C. Petito, Mathew J. Feinstein, Chad J. Achenbach, Janna Williams, Babafemi Taiwo

**Affiliations:** 1 Institute for Public Health and Management, Feinberg School of Medicine, Chicago, IL 60611 USA; 2 Department of Research and Evaluation, Kaiser Permanente Southern California, Pasadena, CA 91101 USA; 3 Division of Infectious Diseases, Department of Medicine, Northwestern University Feinberg School of Medicine, 645 N. Michigan Ave, Suite 900, Chicago, IL 60611 USA; 4 Division of Biostatistics, Department of Preventive Medicine, Feinberg School of Medicine, Chicago, IL 60611 USA; 5 Havey Institute for Global Health, Northwestern University Feinberg School of Medicine, Chicago, IL 606011 USA; 6 Division of Cardiology, Department of Medicine, Feinberg School of Medicine, Chicago, IL 60611 USA; 7 Division of Epidemiology, Department of Preventive Medicine, Northwestern University Feinberg School of Medicine, Chicago, IL USA; 8 Department of Preventive Medicine, Northwestern University Feinberg School of Medicine, Chicago, IL USA

**Keywords:** COVID-19, Statin, Mortality, Critical care, Targeted maximum likelihood estimation, Observational studies

## Abstract

**Importance:**

Statin use prior to hospitalization for Coronavirus Disease 2019 (COVID-19) is hypothesized to improve inpatient outcomes including mortality, but prior findings from large observational studies have been inconsistent, due in part to confounding. Recent advances in statistics, including incorporation of machine learning techniques into augmented inverse probability weighting with targeted maximum likelihood estimation, address baseline covariate imbalance while maximizing statistical efficiency.

**Objective:**

To estimate the association of antecedent statin use with progression to severe inpatient outcomes among patients admitted for COVD-19.

**Design, setting and participants:**

We retrospectively analyzed electronic health records (EHR) from individuals ≥ 40-years-old who were admitted between March 2020 and September 2022 for ≥ 24 h and tested positive for SARS-CoV-2 infection in the 30 days before to 7 days after admission*.*

**Exposure:**

Antecedent statin use—statin prescription ≥ 30 days prior to COVID-19 admission.

**Main outcome:**

Composite end point of in-hospital death, intubation, and intensive care unit (ICU) admission.

**Results:**

Of 15,524 eligible COVID-19 patients, 4412 (20%) were antecedent statin users. Compared with non-users, statin users were older (72.9 (SD: 12.6) versus 65.6 (SD: 14.5) years) and more likely to be male (54% vs. 51%), White (76% vs. 71%), and have ≥ 1 medical comorbidity (99% vs. 86%). Unadjusted analysis demonstrated that a lower proportion of antecedent users experienced the composite outcome (14.8% vs 19.3%), ICU admission (13.9% vs 18.3%), intubation (5.1% vs 8.3%) and inpatient deaths (4.4% vs 5.2%) compared with non-users. Risk differences adjusted for labs and demographics were estimated using augmented inverse probability weighting with targeted maximum likelihood estimation using Super Learner*.* Statin users still had lower rates of the composite outcome (adjusted risk difference: − 3.4%; 95% CI: − 4.6% to − 2.1%), ICU admissions (− 3.3%; − 4.5% to − 2.1%), and intubation (− 1.9%; − 2.8% to − 1.0%) but comparable inpatient deaths (0.6%; − 1.3% to 0.1%).

**Conclusions and relevance:**

After controlling for confounding using doubly robust methods, antecedent statin use was associated with minimally lower risk of severe COVID-19-related outcomes, ICU admission and intubation, however, we were not able to corroborate a statin-associated mortality benefit.

**Supplementary Information:**

The online version contains supplementary material available at 10.1186/s12879-023-08026-0.

## Key points

**Question:**

Is statin use prior to hospital admission for COVID-19 associated with reducing severe inpatient outcomes?

**Findings:**

In this observational study using electronic health records from a multi-hospital health system in Chicago, we used robust statistical methods to account for confounding and found that adults 40 years or older who were prescribed statins prior to admission for COVID-19 had minimally lower rates of intubation and admission to the intensive care unit. However, inpatient mortality was comparable between statins users and non-users.

**Meaning:**

Consistent with current COVID-19 treatment guidelines, we did not find evidence supporting the utilization of statins for clinically significant reduction in severe inpatient COVID-19 outcomes.

**Supplementary Information:**

The online version contains supplementary material available at 10.1186/s12879-023-08026-0.

## Introduction

The Coronavirus Disease 2019 (COVID-19) pandemic continues to cause hospitalizations and deaths worldwide. Over two years into the pandemic, there are very few therapeutic options proven to improve hospital outcomes due to infection with SARS-CoV-2 [1]. Early on, several observational studies posited that statin use could offer some therapeutic benefit to hospitalized COVID-19 patients, such as lower rates of intubation or mortality [2–8]. Additionally, experimental studies revealed potential mechanisms for this benefit by demonstrating that statins may hinder viral entry and viral replication as well as reduce inflammatory response to infection and inhibit pro-thrombotic processes [5, 9–19].

To date, only one randomized trial (INSPIRATION-S, ClinicalTrials.gov Identifier: NCT02735707) has concluded that investigated the effect of statin initiation with hospitalization outcomes. In this trial limited to those admitted in the ICU, atorvastatin use was not associated with any mortality reduction [20]. Due to the limited number of randomized trials of statin use powered to evaluate effects on COVID-19 outcomes and the difficulty of evaluating pre-admission interventions (e.g., antecedent statin use) with trials, observational data offer opportunities to evaluate associations of statin use with COVID-19 outcomes. However, interpretations of such analyses have limitations, particularly related to confounding. Individuals taking statins are generally older and have more comorbidities, which apart from statin use confer higher risks of worse COVID-19 outcomes but may also exhibit higher rates of health protective behavior than non-users [13, 15, 21, 22]. Many observational studies of the associations between statins and COVID-19 outcomes are confounded and have produced conflicting results[23–25]. Meta-analyses of observational studies suggested an overall protective association [26], but pooling results only improves precision of the estimate of the association but cannot address confounding of antecedent statin use and clinical outcomes inherent in the study design [27]. Well-designed observational studies using rigorous analytical approaches can offer timely evidence to support clinical decision making and practice guidelines [28].

In this study, we compared the risk for progression to severe inpatient outcomes between antecedent statin users and non-users who were admitted for COVID-19 across a multi-hospital health system in Chicago. We leveraged nearly two years of data drawn from a diverse patient population and applied state-of-the-art doubly robust methods combined with machine learning techniques to control for confounding by indication while maximizing statistical efficiency [29].

## Methods

### Population and data source

We used the Northwestern Medicine Electronic Data Warehouse (NMEDW) to conduct an observational cohort study. We included in the analysis any adult who was: (i) 40 years or older, (ii) admitted for at least 24 h in any hospital that was part of the Northwestern Medicine Health System from March 1, 2020 to Sep 27, 2022, (iii) tested positive for SARS-CoV-2 between 30 days before and up to seven days after first date of admission and (iv) not admitted for elective procedures, therapy or drug infusions.

This study received ethical approval from the Northwestern University Institutional Review Board (IRB #: STU00212267).

### Exposure

The main exposure of interest was antecedent statin use defined as any statin prescription existing at least 30 days prior to COVID-19 hospital admission. Absence of any recorded statin prescription implied not taking statins. Individuals who began statins 29 days prior to through the end of COVID-19 hospital admission were not considered antecedent statin users; these individuals were still included in our analysis to maintain sample size. As their data hypothetically would bias our results towards the null, we conducted a sensitivity analysis excluding this group (see below).

### Outcomes

The primary outcome for the antecedent exposure was a composite end point with any of three adverse inpatient outcomes: (i) direct admission or transfer to the intensive care unit (ICU), (ii) intubation, and (iii) inpatient death. We also conducted analyses using each outcome separately.

### Potential confounders

We considered the following variables as confounders and associated with both the probability of antecedent statin use and inpatient outcomes based on clinical knowledge of authors and prior studies on this topic: age, gender, body mass index (BMI) 30 or higher, race category, Hispanic ethnicity, SARS-CoV-2 vaccination status at baseline, county of residence, co-morbidities (asthma, cancer, cardiovascular disease, chronic obstructive pulmonary disease, chronic liver disease, diabetes, HIV, hypertension, immune disorders, and renal disease) based on International Classification of Diseases (ICD) codes, and month of admission (see Additional file 1 for list of ICD codes and SARS-CoV-2 vaccine status definition). In sensitivity analysis, we also included variables associated with the outcome but not necessarily antecedent statin use: site of admission, and clinical presentation severity indicators (heart rate, respiratory rate, oxygen saturation, and systolic blood pressure).

### Statistical analysis

Estimating the association between antecedent statin use and inpatient outcomes in observational data becomes obscured by confounding by indication. Antecedent statin users are likely incomparable with non-users across several factors that might increase their risk for adverse inpatient COVID-19 outcomes. For example, people with history of cardiovascular disease are more likely to be put on statins and are more likely to die due to complications of COVID-19. We addressed this incomparability in two ways. First, we restricted the analysis to those 40 years and older to be commensurate with practice guidelines [30], and to ensure that our non-user population was not systematically younger than statin users. Second, we adjusted for non-exchangeability of individuals by using inverse probability weighting (IPW) to adjust for baseline factors. IPW was selected over propensity score matching as it allows for easier interpretation of findings in relation to the original population, and avoids artificially induced selection bias [31].

The usual inverse propensity weighting (IPW) approach uses a main effects multivariable logistic regression model to create weights that represent treatment assignment, and then applies that weight in the outcome model, here a logistic regression model adjusting only for treatment status, to calculate the quantity of interest (an adjusted risk difference). This approach assumes correct specification of the treatment assignment model to interpret estimates as unbiased. Augmented IPW (AIPW), a doubly robust method, further adjusts the outcome model for a priori specified covariates, relaxing this assumption to correct specification of *either* the treatment assignment or outcome models. Here, we combined AIPW with targeted maximum likelihood estimation (TMLE), a state-of-the-art statistical technique, to incorporate machine learning approaches into the model specification for AIPW while maximizing statistical efficiency[32, 33]. This incorporation of flexible functional forms in both the treatment assignment and outcome models maximizes our chance of satisfying assumptions about model specification. In this analysis, we chose to use Super Learner [34], an ensemble-based machine learning technique, to create the treatment assignment and outcome models, as it has demonstrated statistical superiority in the presence of model misspecification [35]. Following guidelines [36], we included five learners that cover many frequently used machine learning techniques: mean, main effects logistic regression, lasso logistic regression, random forest, and gradient boosted trees. Continuous variables were specified as restricted cubic splines to offer maximum flexibility. All analyses were run in R 4.1.0 using the following packages: SuperLearner [34], tmle [33], and AIPW [32].

For sensitivity analysis, we repeated the analysis comparing only those who were antecedent users and never users (people who never started statins even during admission); in theory this sensitivity analysis should be biased away from the null. We also repeated the analysis using the singly-robust IPW approach where weights were either estimated using logistic regression or using gradient boosted trees with the WeighIt package [37].

Finally, we repeated the main analysis using a three-category exposure: high intensity statin, low-to-moderate intensity statin, and non-user, to assess if intensity of treatment was associated with the outcome. High intensity statin was defined as atorvastatin ≥ 40 mg or rosuvastatin ≥ 20 mg and all other statin prescriptions were classified as low-to-moderate intensity, as systematically defined previously [38]. For this sub-analysis, we used classic TMLE (as implemented in tmle3 [39]) because the current AIPW package only supports binary exposures.

## Results

### Overview of the cohort

A total of 19,342 persons were admitted for COVID-19 from March 1, 2020 to September 29, 2021 in the Northwestern Medicine system. After applying our inclusion criteria, we included 15,524 in our analytic sample for the antecedent use analysis. Patients not meeting inclusion criteria tended to be younger, female, Hispanic, and had lower prevalence of comorbidities. (Additional file 2: Table S1).

### Comparison of antecedent users and non-users

In our analytic sample, 4412 (28%) were antecedent statin users and tended to be older, male, white, received at least one COVID-19 vaccine dose, and have a comorbidity such as asthma, hypertension, diabetes, and cardiovascular disease (Table 1). Balance between groups was achieved by using weights estimated using a stacked learner. Alternative weights estimators also achieved balance (Additional file 3: Figures S1-S3).

**Table 1:** Demographics and Clinical Characteristics at COVID-19 Admission of the Analytical Cohort, Northwestern Medical Group, March 2020-September 2022

	Non-antecedent statin user (n = 11,112)	Antecedent statin user (n = 4,412)
Baseline variables		
Age, mean (SD)	65.56 (14.53)	72.09 (12.62)
Male sex, n (%)	5635 (50.7)	2386 (54.1)
Race, n (%)		
Asian	338 (3.1)	127 (2.9)
Black	1468 (13.5)	606 (13.9)
Multiracial^1^	198 (1.8)	53 (1.2)
White	7767 (71.3)	3329 (76.3)
Other^2^	1117 (10.3)	248 (5.7)
Hispanic/Latino/x ethnicity, n (%)	1927 (17.3)	367 (8.3)
County, n (%)		
Cook	3033 (28.4)	1227 (28.2)
DeKalb	760 (7.1)	419 (9.6)
DuPage	1835 (17.2)	722 (16.6)
Kane	1044 (9.8)	508 (11.7)
Lake	1622 (15.2)	489 (11.2)
McHenry	1629 (15.3)	744 (17.1)
Others in Illinois^3^	374 (3.5)	147 (3.4)
Non-Illinois Areas	375 (3.5)	93 (2.1)
Site		
1	2199 (19.8)	848 (19.2)
2	931 (8.4)	487 (11.0)
3	1153 (10.4)	395 (9.0)
4	718 (6.5)	371 (8.4)
5	1488 (13.4)	435 (9.9)
6	1221 (11.0)	605 (13.7)
7	3212 (28.9)	1211 (27.4)
8, 9 or 10	190 (1.7)	60 (1.4)
BMI ≥ 25, n (%)	7336 (73.5)	2908 (73.2)
Ever smoked, n (%)	2311 (20.8)	662 (15.0)
Vaccination status on admission, n (%)		
None	8656 (77.9)	2353 (53.3)
Incomplete	1197 (10.8)	960 (21.8)
Full	222 (2.0)	162 (3.7)
Boosted	1037 (9.3)	937 (21.2)
Quarter and Year of Admission, n (%)		
2020 Quarter 1	249 (2.2)	10 (0.2)
2020 Quarter 2	1456 (13.1)	143 (3.2)
2020 Quarter 3	512 (4.6)	107 (2.4)
2020 Quarter 4	2384 (21.5)	726 (16.5)
2021 Quarter 1	978 (8.8)	411 (9.3)
2021 Quarter 2	623 (5.6)	194 (4.4)
2021 Quarter 3	607 (5.5)	233 (5.3)
2021 Quarter 4	1224 (11.0)	547 (12.4)
2022 Quarter 1	1335 (12.0)	745 (16.9)
2022 Quarter 2	679 (6.1)	503 (11.4)
2022 Quarter 3	835 (7.5)	636 (14.4)
2022 Quarter 4	230 (2.1)	157 (3.6)
Initial Clinical Presentation, mean (SD)		
Heart Rate (per minute),	90.30 (19.09)	86.82 (19.74)
Respiratory rate (per minute)	21.09 (5.76)	20.66 (5.18)
Systolic blood pressure (mmHg)	136.33 (25.19)	137.46 (26.81)
Diastolic blood pressure (mmHg)	75.10 (14.38)	72.96 (14.80)
Oxygen Saturation (%)	94.54 (6.22)	95.08 (4.81)
Comorbidities at baseline, n (%)		
Asthma	1396 (12.6)	825 (18.7)
Cancer	3068 (27.6)	1852 (42.0)
Cardiovascular disease	5152 (46.4)	3537 (80.2)
Chronic liver disease	449 (4.0)	243 (5.5)
COPD	1195 (10.8)	1005 (22.8)
Cardiovascular disease	5152 (46.4)	3537 (80.2)
Diabetes mellitus	3356 (30.2)	2397 (54.3)
HIV	260 (2.3)	196 (4.4)
Hypertension	6902 (62.1)	4013 (91.0)
Immune disorder	1071 (9.6)	708 (16.0)
Renal disease	3227 (29.0)	2413 (54.7)
Treatment during admission, n (%)		
Bamlanivumab	10 (0.1)	12 (0.3)
Dexamethasone	5368 (48.3)	2120 (48.1)
Remdesivir	4438 (39.9)	2006 (45.5)
Sarilumab	15 (0.1)	1 (0.0)
Tocilizumbab	512 (4.6)	96 (2.2)
Steroids	6221 (56.0)	2532 (57.4)
Immune modulator^4^	401 (3.6)	285 (6.5)
Outcomes		
Composite inpatient outcome, n (%)	2140 (19.3)	653 (14.8)
ICU admission, n (%)	2034 (18.3)	614 (13.9)
Intubation, n (%)	919 (8.3)	226 (5.1)
Inpatient death, n (%)	583 (5.2)	196 (4.4)
Time to composite outcome, median [IQR]	0.31 [0.14, 2.69]	0.41 [0.18, 3.54]
Length of Stay, median [IQR]	4.88 [2.80, 8.84]	4.51 [2.81, 7.89]

BMI: body mass index, COPD: chronic obstructive pulmonary disease

^1^Multiracial individuals are people who report more than two categories (e.g., Asian and Black)

^2^Others include American Indian, Alaska Native, Native Hawaiian, Pacific-Islander, Guamanian, and Chamorro or chose other or none of the above

^3^Other Illinois counties include all other counties not listed in the table

^4^See methods supplement for complete list of immune-modulator drugs. Variables with missing data: Body mass index (10%), county (3%), race (2%), insurance (0.1%), Systolic blood pressure (0.05%), Diastolic blood pressure (0.05%), Oxygen saturation (0.03%)

In unadjusted analyses, a significantly lower proportion of statin users experienced the composite outcome (14.8% vs 19.3%, p < 0.001). The component events were also significantly lower in statin users: ICU admission (13.9% vs 18.3%, p < 0.001), intubation (5.1% vs 8.3%, p < 0.001), and inpatient deaths (4.4% vs 5.2%, p = 0.04) (Table 1).

### Main analysis

Using AIPW with TMLE and stacked learners, we found that antecedent statin users had a minimal, but significantly lower risk of the composite outcome (Risk difference (RD): − 3.2, 95% CI: − 4.6 to − 1.9) after adjusting for baseline covariates. Per outcome analysis, we found that antecedent statin users had significantly lower risk of ICU admission (RD: -3.2, 95%CI: − 4.5 to − 1.8) and intubation (RD: − 1.6, 95%CI: − 2.4 to − 0.80). However, risk of inpatient death (RD: − 0.40, 95%CI: − 1.2 to 0.3) were comparable between the two groups. Conclusions remained roughly the same even after adding site of admission (i.e., which hospital within the system) and clinical presentation on admission in the models (Table 2).

**Table 2:** Adjusted risk differences for adverse inpatient outcomes between antecedent statin users and non-users, Northwestern Medical Group, March 2020-September 2022

Adverse Inpatient Outcomes	Unadjusted Rate per 100 admitted patients (%)		Risk difference^1^ (%) (95% CI)			
	Antecedent statin users (n = 4,412)	Non-users (n = 11,112)	Unadjussted	Baseline	Baseline + Site	Baseline + Site + Clinical
Composite outcome	14.8	19.3	− 4.5 (− 5.7, − 3.2)*	− 3.4 (− 4.6, − 2.1)*	− 3.3 (− 4.5, − 2.1)*	− 2.4 (− 3.4, − 1.5)*
Intensive care unit admission	13.9	18.3	− 4.4 (− 5.6, − 3.1)*	− 3.3 (− 4.5, − 2.1)*	− 3.2 (− 4.4, − 2.0)*	− 2.4 (− 3.4, − 1.4)*
Intubation	5.1	8.3	− 3.2 (− 4.0, − 2.3)*	− 1.9 (− 2.8, − 1.0)*	− 1.7 (− 2.5, − 0.9)*	− 1.3 (− 2.0, − 0.7)*
Inpatient death	4.4	5.2	− 0.8 (− 1.5, − 0.07)*	− 0.6 (− 1.3, 0.1)	− 0.5 (− 1.2, 0.1)	− 0.4 (− 0.9, 0.1)

^*^ − 95% confidence interval lies on the same side of null (zero) suggesting significant difference between the two groups. 1—Risk difference calculated as risk among statin users minus risk among non-users. Unadjusted risk difference is based on an unweighted logistic regression model with just exposure status as a covariate. Baseline covariates include age, gender, body mass index 25 or higher (binary), race, Hispanic ethnicity, county of residence, co-morbidities (asthma, cancer, cardiovascular disease, chronic obstructive pulmonary disease, chronic liver disease, diabetes, hypertension, and renal disease), and month of admission. Site variables are dummies for hospital facility and clinical variables include baseline measures of heart rate, respiratory rate, systolic and diastolic blood pressure, and oxygen saturation

### Sensitivity analysis

Findings were robust to sensitivity analysis. Using an approach where weights were derived from logistic regression or boosted trees also did not lead to materially different conclusions. (Additional file 2: Table S2) Findings were also similar if the analysis was restricted to antecedent users and never users (i.e., did not start statins during admission) although the differences were smaller (e.g., RDcomp: − 1.9, 95%CI: − 3.5 to − 0.2). (Additional file 2: Table S3).

Exploratory analysis using a three-category exposure showed that non-users had higher estimated rates of the composite outcome, ICU admission, and intubation with no apparent dose–response curve (i.e., declining risk with increasing intensity) for these outcomes. Interestingly, high intensity users were found to have significantly lower inpatient mortality compared to non-users while the low-to-moderate intensity users had comparable mortality risk versus non-users (Table 3).

**Table 3:** Adjusted rates and risk differences for adverse inpatient outcomes according to statin intensity, Northwestern Medical Group, March 2020-September 2022

Outcome	Estimated Rate per 100 admitted Patients (95% CI)			Risk Difference (vs non-user) (95% CI)
A. Adjusting Baseline covariates only				
Composite				
Non-user	18.84 (18.07, 19.61)			NA
Low-moderate intensity	15.17 (13.34, 17)			− 3.67 (− 5.65, − 1.7)*
High intensity	15.96 (13.84, 18.08)			− 2.88 (− 5.12, − 0.63)*
ICU admission				
Non-user	17.89 (17.14, 18.64)			NA
Low-moderate intensity	14.09 (12.32, 15.87)			− 3.8 (− 5.72, − 1.87)*
High intensity	15.5 (13.42, 17.58)			− 2.39 (− 4.6, − 0.18)*
Intubation				
Non-user	7.76 (7.24, 8.27)			NA
Low-moderate intensity	6.02 (4.73, 7.31)			− 1.74 (− 3.11, − 0.36)*
High intensity	5.68 (4.31, 7.06)			− 2.07 (− 3.54, − 0.61)*
Inpatient Death				
Non-user	5.14 (4.71, 5.57)			NA
Low-moderate intensity	4.7 (3.77, 5.62)			− 0.44 (− 1.45, 0.56)
High intensity	3.93 (2.95, 4.91)			− 1.21 (− 2.28, − 0.15)*
B. Adjusting for Baseline and site variables				
Composite				
Non-user	18.83 (18.07, 19.6)			NA
Low-moderate intensity	15.14 (13.35, 16.92)			− 3.7 (− 5.63, − 1.77)*
High intensity	15.9 (13.82, 17.98)			− 2.93 (− 5.14, − 0.72)*
ICU admission				
Non-user	17.88 (17.13, 18.63)			NA
Low-moderate intensity	14.23 (12.49, 15.97)			− 3.65 (− 5.53, − 1.76)*
High intensity	15.67 (13.58, 17.77)			− 2.21 (− 4.43, 0.02)
Intubation				
Non-user	7.71 (7.2, 8.21)			NA
Low-moderate intensity	6.06 (4.79, 7.33)			− 1.65 (− 3.01, − 0.28)*
High intensity	5.68 (4.34, 7.03)			− 2.02 (− 3.46, − 0.59)*
Inpatient Death				
Non-user	5.11 (4.69, 5.54)			NA
Low-moderate intensity	4.79 (3.84, 5.74)			− 0.33 (− 1.36, 0.7)
High intensity	3.87 (2.93, 4.82)			− 1.24 (− 2.27, − 0.21)*
C. Adjusting for Baseline, site, and clinical variables				
Composite				
Non-user	18.64 (17.89, 19.39)			NA
Low-moderate intensity	15.53 (13.72, 17.34)			− 3.11 (− 5.05, − 1.17)*
High intensity	16.01 (14, 18.03)			− 2.62 (− 4.75, − 0.5)*
ICU admission				
Non-user	17.88 (17.13, 18.62)			NA
Low-moderate intensity	14.13 (12.39, 15.88)			− 3.74 (− 5.63, − 1.85)*
High intensity	15.47 (13.4, 17.54)			− 2.4 (− 4.59, − 0.22)*
Intubation				
Non-user	7.71 (7.2, 8.22)			NA
Low-moderate intensity	6.21 (4.88, 7.53)			− 1.5 (− 2.91, − 0.09)*
High intensity	5.65 (4.31, 6.98)			− 2.06 (− 3.49, − 0.64)*
Inpatient Death				
Non-user	5.09 (4.66, 5.51)			NA
Low-moderate intensity	4.82 (3.88, 5.77)			− 0.26 (− 1.29, 0.76)
High intensity	3.88 (2.95, 4.82)			− 1.2 (− 2.22, − 0.19)*

CI – confidence interval *—95% CI lies on the same side of null (zero) suggesting significant difference between the two groups. High intensity statin was defined as atorvastatin ≥ 40 mg or rosuvastatin ≥ 20 mg. Any other prescription was considered low-to-moderate intensity. Assessment was based on the prescription closest to 30 days before the admission of interest. Sample size for each group are as follows: 11,112 non-users, 2657 low-to-moderate intensity users, and 1755 high intensity users. Baseline covariates include age, gender, body mass index (BMI) 25 or higher (binary), race, Hispanic ethnicity, county of residence, co-morbidities (asthma, cancer, cardiovascular disease, chronic obstructive pulmonary disease, chronic liver disease, diabetes, hypertension, and renal disease), and month of admission. Site variables are dummies for hospital facility and clinical variables include baseline measures of heart rate, respiratory rate, systolic and diastolic blood pressure, and oxygen saturation

## Discussion

Using state-of-the-art doubly robust techniques and 31 months of pandemic data, we observed that antecedent statin use was associated with minimally lower rates of the composite COVID-19 hospital outcome of ICU transfer, intubation, or inpatient death The observed difference between antecedent users and non-users was driven by the difference in ICU admission and intubation rather than death. Importantly, the clinical significance of these small differences were minimal. Compared to other published studies, an early large observational study concluded that antecedent statin use was associated with lower risk of adverse outcomes, including death [4]. However, other large studies conducted in Denmark, Italy, and Spain have failed to detect such benefit [23–25]. Meta-analyses on this issue suggested that there may be a mortality benefit with antecedent statin use [26] although one of the meta-analyses [25] pooled studies that examined both antecedent and in-patient statin use. While our work differs with the INSPIRATION-S trial in the timing of statin use, our findings are complementary to INSPIRATION-S which found no mortality benefits from statin initiation during ICU admission [20].

Statins were considered for mitigating COVID-19 severity due to their known effect on cholesterol levels. Cholesterol has been linked to proinflammatory effects that were hypothesized may lead to poor COVID-19 outcomes due to activation of macrophages and dysregulation of NF-κB and TNF alpha pathways [5, 10, 16–18]. Furthermore, cholesterol has also been associated with the viral entry process [11, 12, 15, 19]. By reducing cholesterol, statins were hypothesized that statins would lead to less severe disease. This is in addition to any improvements in cardiovascular health from statin exposure [40]. Had these biologic effects been confirmed clinically or epidemiologically, statins might have been seen as good treatment candidates given their low cost (compared to antivirals) and availability even in low-resource settings. However, our findings related to inpatient mortality suggested that the presumed effects of statins, if present, are likely small and high intensity statins may be needed to be clinically apparent.

In addition, unmeasured confounders could also explain the benefits we observed in the antecedent statin analysis. Antecedent statin use could be a marker of unmeasured confounding related to care differences or lower severity of COVID-19 at hospital admission and, thus lower probability of requiring ICU level care. For example, since patients on statins are older and with cardiovascular co-morbidities or risk factors [41], clinicians may have admitted those with milder clinical disease for hospital observation or to receive intravenous anti-viral therapy (remdesivir) due to concerns about risk of progression to more severe COVID-19 [42, 43]. If true, such practice might have paradoxically led to lower rates of progression to ICU admission among statin users as observed in the unadjusted results. Thus, after using doubly robust methods to better control for confounding, antecedent statin use was associated with minimal, but significant lower risk of severe COVID-19-related outcomes of ICU admission and intubation; however, we were not able to corroborate a statin-associated mortality benefit found by prior studies. Therefore, our study does not provide evidence to support utilization of antecedent statins to improve inpatient COVID-19 outcomes; this is consistent with current COVID-19 treatment guidelines [1].

The methodological approach in this study has some key differences from prior work that could explain our robust findings [44, 45]. First, this study period covered almost 24 months, which allowed us to capture several stages of the pandemic, including the period after introduction of vaccines and the Omicron wave in mid-2022. Our analysis thus evaluated antecedent statin use across a wide context of care and several waves of virus variants. Second, we restricted our exposure to antecedent use. This helps us ensure a consistent exposure unlike other studies where both inpatient initiation and antecedent use were grouped together as statin users. Third, we used methods that are less susceptible to misspecification of the weights and outcome models. While many studies utilized weighting or matching to address confounding [45], none used doubly robust techniques making their approach susceptible to bias from model misspecification. Finally, we chose weighting over matching. While the issue of weighting versus matching is widely discussed in the causal inference literature [31], we chose weighting because it retains as much of the sample as possible and preserves interpretability of our findings to the original population.

This work has several key limitations. First is that we used the EHR to measure outcomes, exposures, and covariates as such were limited to data recorded in the EHR. This could have resulted in misclassification of exposure where an individual’s use or non-use of statins might have been misclassified if their records had not been updated. Further, we assumed that patients identified as being on a statin in the EHR were taking this medication whereas it is possible for patients to be poorly adherent to prescribed drugs. We could have also misclassified some diagnoses of comorbidities due to reliance solely on ICD codes [46]. Second, despite our best efforts and novel methods, we cannot fully account for unmeasured confounding and healthy-user bias. Healthy-user bias suggests that people who are on antecedent statins will be healthier than people who are not on statins due to higher rates of health protective behavior. For example, statin users were compliant with pneumococcal vaccination [47]. We observed this in our data as well with antecedent statin users more likely to be vaccinated for COVID-19 by time of admission. Meanwhile, confounding by indication suggest that those on statins had worse health than people who were not on statins because they do not need to be on statins and had better cardiovascular health profiles. These are pathways that potentially bias the estimation in different and diverging directions (48). If healthy-user bias was more dominant, we would expect significantly better outcomes in the treated group. This could be the case only for ICU admission and intubation but not deaths in the main analysis. This could also explain the mortality benefit from high intensity statin use. Unfortunately, our use of EHR data alone limit our ability to thoroughly address these questions without availability of supplemental information on pre-COVID-19 health and behavior. Variables such as lipid levels could be proxies, however, the lipids variables were available differentially (nearly absent in non-users) and we therefore judged inclusion of lipids in modeling would likely add bias to the study rather than mitigate bias. Third, is that we limited our analysis to adults at least 40 years old since we observed very little antecedent statin users in younger individuals. While it helped improve balance, it also limited generalizability to younger adults and comparability to other studies that did not impose this criterion. Finally, we can only measure association and not the causal effect of antecedent statin use on inpatient outcomes. To measure a causal effect requires emulation of a target trial which is not feasible with the dataset given that we only have complete data among those who were subsequently admitted for COVID-19.

In conclusion, using our EHR we performed a clinical cohort study to evaluate the association of antecedent statin use with key severe outcomes of people hospitalized for COVID-19 after carefully adjusting for confounding. Using state of the art doubly robust methods, our data suggests that antecedent statin users had a minimal, but significantly lower risk of the composite outcome. Upon further analysis, the minimal reduction in composite outcome risk was driven mostly by ICU admission or intubation and not by a reduction in mortality, contrary to some previous studies. Furthermore, these results were not affected by statin dose intensity. This study highlights the complexities and urges caution when using observational clinical care data to investigate associations that may be confounded by multiple measured and unmeasured factors. While our findings indicate a lack of mortality benefit in hospitalized COVID-19 patients, we found no evidence of harm. Thus, although our observations were insufficient to strongly recommend for or against statin use specifically related to COVID-19, statin use should still of course be encouraged for conditions where it has a primary indication such as atherosclerotic cardiovascular disease primary and secondary prevention.

## Supplementary Information

**Additional file 1. **Additional Methods. Sections: A. Operational definitions of comorbidities and vaccine status. B. Missing data imputation C. Augmented Inverse Propensity Weighting with Targeted Maximum Likelihood Estimation. D. TMLE for three-category exposures. E. Sensitivity analyses for antecedent statin use using inverse propensity scores.

**Additional file 2: Table S1.** Demographic and Clinical Characteristics of Included versus Excluded Admissions, Northwestern Medical Group, March 2020-September 2022. **Table S2.** Sensitivity Analysis for Risk Differences of Inpatient Outcomes using Outcome Regression and Inverse Propensity Weighting, Northwestern Medical Group, March 2020-September 2022. **Table S3.** Sensitivity Analysis for Antecedent Statin and Risk Differences of Inpatient Outcomes Comparing only Never users to Antecedent users, Northwestern Medical Group, March 2020-September 2022.

**Additional file 3: Figure S1.** Love plots for covariate balance using different weighting methods with baseline variables, Northwestern Medical Group, March 2020-September 2022. **Figure S2.** Love plots for covariate balance using different weighting methods with baseline and site variables, Northwestern Medical Group, March 2020-September 2022. **Figure S3.** Love plots for covariate balance using different weighting methods with baseline, site and clinical variables, Northwestern Medical Group, March 2020-September 2022.

## Data Availability

Because of the sensitive nature of the data analyzed for this study, requests to access the dataset from qualified researchers trained in human subject confidentiality protocols may be sent to first author (OA) or co-author (CA).
