# Supplementary material for: Association of statin use with outcomes of patients admitted with COVID-19: an analysis of electronic health records using superlearner
Source: BMC Infect Dis. 2023 Feb 24;23:115. doi: 10.1186/s12879-023-08026-0 (PMC9951166; doi:10.1186/s12879-023-08026-0)
Supplement: Supplementary file 1 — Additional file 1. Additional Methods. Sections: A. Operational definitions of comorbidities and vaccine status. B. Missing data imputation C. Augmented Inverse Propensity Weighting with Targeted Maximum Likelihood Estimation. D. TMLE for three-category exposures. E. Sensitivity analyses for antecedent statin use using inverse propensity scores. [file 12879_2023_8026_MOESM1_ESM.docx]

**Additional File 1. Supplemental Methods**

Association of Statin Use with Outcomes of Patients Admitted with COVID-19: An Analysis of Electronic Health Records using Superlearner

Adovich S. Rivera MD*^1,2^, Omar Al-Heeti MD*^3^, Lucia C. Petito PhD^4^, Mathew J. Feinstein MD MS^4,6^, Chad J. Achenbach MD, MPH^3,4,5^, Janna Williams MD^3^, Babafemi Taiwo MBBS^3,5^

^1^Institute for Public Health and Management, Feinberg School of Medicine, Chicago, IL, 60611

2Department of Research and Evaluation, Kaiser Permanente Southern California, Pasadena, CA, 91101

^3^Department of Medicine, Division of Infectious Diseases, Northwestern University Feinberg School of Medicine, Chicago, IL 60611

^4^Department of Preventive Medicine, Division of Biostatistics, Feinberg School of Medicine, Chicago, IL, 60611

^5^Havey Institute for Global Health, Northwestern University Feinberg School of Medicine, Chicago, IL 606011

^6^Department of Medicine, Division of Cardiology, Feinberg School of Medicine, Chicago, IL, 60611

^*co-first author^

**Sections**

A. Operational definitions of comorbidities and vaccine status

B. Missing data imputation

C. Augmented Inverse Propensity Weighting with Targeted Maximum Likelihood Estimation

D. TMLE for three-category exposures

E. Sensitivity analyses for antecedent statin use using inverse propensity scores

1. **Operational definitions of comorbidities and vaccine status**
   1. Comorbidities were identified using the following ICD codes

| Comorbidity | ICD codes |
| --- | --- |
| Asthma | 493, 493.0, 493.00, 493.01, 493.02, 493.10, 493.11, 493.12, 493.20, 493.21, 493.22, 493.81, 493.82, 493.9, 493.90, 493.91, 493.92, J45.20, J45.21, J45.22, J45.30, J45.31, J45.32, J45.40, J45.41, J45.42, J45.50, J45.51, J45.52, J45.901, J45.902, J45.909, J45.99, J45.990, J45.991, J45.998 |
| Cancer | 140.1, 141.0, 141.1, 141.2, 141.3, 141.4, 141.6, 141.8, 141.9, 142.0, 142.1, 142.9, 144.0, 144.8, 144.9, 145.0, 145.2, 145.3, 145.5, 145.9, 146.0, 146.1, 146.2, 146.6, 146.9, 147.1, 147.2, 147.8, 147.9, 148.1, 148.3, 148.9, 149.0, 149.8, 149.9, 150.0, 150.2, 150.3, 150.4, 150.5, 150.8, 150.9, 151.0, 151.2, 151.4, 151.5, 151.6, 151.8, 151.9, 152, 152.0, 152.3, 152.9, 153.0, 153.1, 153.2, 153.3, 153.4, 153.5, 153.6, 153.7, 153.8, 153.9, 154, 154.0, 154.1, 154.2, 154.3, 154.8, 155.0, 155.1, 155.2, 156.0, 156.1, 156.2, 157.0, 157.1, 157.2, 157.3, 157.4, 157.8, 157.9, 158.0, 158.8, 158.9, 159.0, 159.1, 159.9, 160, 160.0, 160.2, 160.9, 161.0, 161.1, 161.8, 161.9, 162.0, 162.2, 162.3, 162.4, 162.5, 162.8, 162.9, 163.9, 164.0, 164.3, 170.0, 170.1, 170.2, 170.4, 170.7, 170.8, 170.9, 171.0, 171.2, 171.3, 171.4, 171.5, 171.6, 171.8, 171.9, 172.0, 172.2, 172.3, 172.4, 172.5, 172.6, 172.7, 172.8, 172.9, 173.0, 173.00, 173.01, 173.02, 173.1, 173.10, 173.11, 173.12, 173.2, 173.20, 173.21, 173.22, 173.3, 173.30, 173.31, 173.32, 173.39, 173.4, 173.40, 173.41, 173.42, 173.49, 173.5, 173.50, 173.51, 173.52, 173.6, 173.60, 173.61, 173.62, 173.69, 173.7, 173.70, 173.71, 173.72, 173.81, 173.82, 173.9, 173.90, 173.91, 173.92, 173.99, 174.0, 174.1, 174.2, 174.3, 174.4, 174.5, 174.6, 174.8, 174.9, 175.9, 176, 176.0, 176.1, 176.8, 176.9, 179, 180.0, 180.1, 180.8, 180.9, 182.0, 182.8, 183.0, 183.2, 183.3, 183.8, 183.9, 184.0, 184.1, 184.3, 184.4, 184.8, 184.9, 185, 186, 186.0, 186.9, 187.2, 187.4, 187.8, 188.0, 188.1, 188.2, 188.3, 188.4, 188.5, 188.6, 188.7, 188.8, 188.9, 189.0, 189.1, 189.2, 189.3, 189.8, 189.9, 190.0, 190.1, 190.6, 190.9, 191.0, 191.1, 191.2, 191.3, 191.4, 191.5, 191.6, 191.7, 191.8, 191.9, 192.0, 192.1, 192.2, 192.3, 192.9, 193, 194.0, 194.1, 194.6, 195.0, 195.1, 195.2, 195.3, 195.4, 195.8, 196.0, 196.1, 196.2, 196.3, 196.5, 196.6, 196.9, 197.0, 197.1, 197.2, 197.4, 197.5, 197.6, 197.7, 197.8, 198.0, 198.1, 198.2, 198.3, 198.4, 198.5, 198.6, 198.7, 198.81, 198.82, 198.89, 199.0, 199.1, 199.2, 200.00, 200.01, 200.02, 200.03, 200.04, 200.05, 200.10, 200.11, 200.13, 200.14, 200.18, 200.20, 200.21, 200.22, 200.23, 200.25, 200.26, 200.27, 200.28, 200.3, 200.30, 200.33, 200.34, 200.38, 200.40, 200.41, 200.42, 200.43, 200.44, 200.45, 200.46, 200.48, 200.50, 200.51, 200.6, 200.60, 200.61, 200.64, 200.65, 200.68, 200.70, 200.72, 200.73, 200.75, 200.78, 200.80, 200.81, 200.83, 200.84, 200.87, 200.88, 201, 201.00, 201.10, 201.40, 201.43, 201.45, 201.46, 201.48, 201.50, 201.51, 201.52, 201.53, 201.56, 201.58, 201.60, 201.62, 201.66, 201.76, 201.90, 201.91, 201.92, 201.95, 201.96, 201.98, 202.00, 202.01, 202.02, 202.03, 202.04, 202.05, 202.06, 202.08, 202.1, 202.10, 202.18, 202.20, 202.28, 202.30, 202.33, 202.34, 202.40, 202.41, 202.45, 202.48, 202.60, 202.70, 202.71, 202.73, 202.78, 202.80, 202.81, 202.82, 202.83, 202.84, 202.85, 202.86, 202.87, 202.88, 202.90, 203.0, 203.00, 203.01, 203.02, 203.10, 203.11, 203.12, 203.80, 204.00, 204.01, 204.02, 204.10, 204.11, 204.12, 204.80, 204.81, 204.82, 204.90, 205.00, 205.01, 205.02, 205.10, 205.11, 205.12, 205.30, 205.31, 205.32, 205.80, 205.82, 205.90, 206.00, 207.10, 207.11, 207.20, 207.80, 208.00, 208.01, 208.02, 208.10, 208.11, 208.21, 208.80, 208.81, 208.90, 208.91, 208.92, 209.00, 209.03, 209.10, 209.17, 209.20, 209.21, 209.23, 209.29, 209.30, 209.31, 209.34, 209.36, 209.40, 209.43, 209.57, 209.60, 209.61, 209.63, 209.69, 209.70, 209.71, 209.72, 209.73, 209.74, 209.75, 209.79, 230.0, 230.1, 230.3, 230.4, 230.5, 230.6, 231.0, 231.9, 232.0, 232.2, 232.3, 232.4, 232.5, 232.6, 232.7, 232.9, 233.0, 233.1, 233.3, 233.30, 233.31, 233.32, 233.39, 233.4, 233.7, 233.9, 234.8, 234.9, 235.0, 235.1, 235.2, 235.3, 235.4, 235.5, 235.6, 235.7, 235.8, 235.9, 236.0, 236.2, 236.3, 236.5, 236.7, 236.90, 236.91, 237.0, 237.1, 237.2, 237.3, 237.4, 237.5, 237.6, 237.70, 237.71, 237.72, 238.0, 238.1, 238.2, 238.3, 238.4, 238.5, 238.6, 238.7, 238.71, 238.72, 238.73, 238.74, 238.75, 238.76, 238.77, 238.79, 238.8, 238.9, 239.0, 239.1, 239.2, 239.3, 239.4, 239.5, 239.6, 239.7, 239.81, 239.89, 239.9, C00.0, C00.1, C00.3, C00.9, C01, C02.0, C02.1, C02.2, C02.3, C02.4, C02.8, C02.9, C04.0, C04.1, C04.8, C04.9, C05.0, C05.1, C05.8, C05.9, C06.0, C06.9, C07, C08.0, C08.9, C09.0, C09.1, C09.8, C09.9, C10.1, C10.2, C10.8, C10.9, C11.0, C11.1, C11.2, C11.3, C11.8, C11.9, C12, C13.2, C13.8, C13.9, C14.0, C14.8, C15.3, C15.4, C15.5, C15.8, C15.9, C16, C16.0, C16.1, C16.2, C16.3, C16.4, C16.5, C16.8, C16.9, C17.0, C17.1, C17.2, C17.3, C17.9, C18.0, C18.1, C18.2, C18.3, C18.4, C18.5, C18.6, C18.7, C18.8, C18.9, C19, C20, C21.0, C21.1, C21.8, C22.0, C22.1, C22.3, C22.7, C22.8, C22.9, C23, C24.0, C24.1, C24.8, C24.9, C25.0, C25.1, C25.2, C25.3, C25.4, C25.7, C25.8, C25.9, C26.0, C26.1, C26.9, C30.0, C31.0, C31.1, C31.8, C31.9, C32, C32.0, C32.1, C32.3, C32.8, C32.9, C33, C34.00, C34.01, C34.02, C34.10, C34.11, C34.12, C34.2, C34.30, C34.31, C34.32, C34.80, C34.81, C34.82, C34.90, C34.91, C34.92, C37, C38.2, C38.3, C38.4, C38.8, C39.0, C40.00, C40.01, C40.02, C40.20, C40.21, C40.22, C40.82, C40.90, C41.0, C41.1, C41.2, C41.3, C41.4, C41.9, C43.0, C43.20, C43.21, C43.22, C43.30, C43.31, C43.39, C43.4, C43.51, C43.59, C43.60, C43.61, C43.62, C43.70, C43.71, C43.72, C43.8, C43.9, C44.00, C44.01, C44.02, C44.09, C44.101, C44.102, C44.111, C44.112, C44.1122, C44.119, C44.1191, C44.1192, C44.121, C44.122, C44.129, C44.199, C44.201, C44.202, C44.209, C44.211, C44.212, C44.219, C44.221, C44.222, C44.229, C44.300, C44.301, C44.309, C44.310, C44.311, C44.319, C44.320, C44.321, C44.329, C44.390, C44.391, C44.399, C44.40, C44.41, C44.42, C44.49, C44.501, C44.509, C44.51, C44.511, C44.519, C44.520, C44.529, C44.599, C44.602, C44.609, C44.611, C44.612, C44.619, C44.621, C44.622, C44.629, C44.691, C44.699, C44.701, C44.702, C44.709, C44.711, C44.712, C44.719, C44.721, C44.722, C44.729, C44.792, C44.799, C44.81, C44.82, C44.90, C44.91, C44.92, C44.99, C45.0, C45.1, C45.7, C45.9, C46.0, C46.1, C46.7, C46.9, C47.11, C47.12, C47.21, C47.3, C47.5, C47.6, C47.9, C48.0, C48.1, C48.2, C48.8, C49.0, C49.10, C49.11, C49.12, C49.20, C49.21, C49.22, C49.3, C49.4, C49.5, C49.6, C49.8, C49.9, C49.A0, C49.A2, C49.A3, C49.A9, C50.011, C50.012, C50.019, C50.111, C50.112, C50.119, C50.211, C50.212, C50.219, C50.221, C50.311, C50.312, C50.319, C50.411, C50.412, C50.419, C50.511, C50.512, C50.519, C50.611, C50.612, C50.619, C50.811, C50.812, C50.819, C50.822, C50.9, C50.911, C50.912, C50.919, C50.921, C50.929, C51, C51.0, C51.1, C51.2, C51.8, C51.9, C52, C53.0, C53.1, C53.8, C53.9, C54.0, C54.1, C54.2, C54.3, C54.8, C54.9, C55, C56.1, C56.2, C56.3, C56.9, C57.00, C57.01, C57.02, C57.4, C57.7, C57.8, C57.9, C60.1, C60.9, C61, C62.00, C62.01, C62.10, C62.11, C62.12, C62.90, C62.91, C62.92, C63.10, C63.2, C64.1, C64.2, C64.9, C65.1, C65.2, C65.9, C66.1, C66.2, C66.9, C67, C67.0, C67.1, C67.2, C67.3, C67.4, C67.5, C67.6, C67.7, C67.8, C67.9, C68.0, C68.8, C68.9, C69.30, C69.31, C69.32, C69.40, C69.42, C69.51, C69.60, C69.61, C69.90, C69.92, C70.0, C70.1, C70.9, C71.0, C71.1, C71.2, C71.3, C71.4, C71.5, C71.6, C71.7, C71.8, C71.9, C72.0, C72.1, C72.30, C72.42, C72.59, C72.9, C73, C74.10, C74.11, C74.90, C74.91, C74.92, C75.0, C75.1, C75.5, C75.9, C76.0, C76.1, C76.2, C76.3, C76.41, C76.42, C76.51, C76.52, C76.8, C77.0, C77.1, C77.2, C77.3, C77.4, C77.5, C77.8, C77.9, C78.00, C78.01, C78.02, C78.1, C78.2, C78.39, C78.4, C78.5, C78.6, C78.7, C78.80, C78.89, C79.00, C79.01, C79.02, C79.10, C79.11, C79.19, C79.2, C79.31, C79.32, C79.40, C79.49, C79.51, C79.52, C79.60, C79.61, C79.62, C79.63, C79.70, C79.71, C79.72, C79.81, C79.82, C79.89, C79.9, C80.0, C80.1, C80.2, C81.00, C81.01, C81.02, C81.03, C81.05, C81.06, C81.08, C81.09, C81.10, C81.11, C81.12, C81.13, C81.14, C81.15, C81.16, C81.18, C81.19, C81.20, C81.22, C81.26, C81.29, C81.30, C81.36, C81.40, C81.45, C81.70, C81.71, C81.72, C81.73, C81.74, C81.76, C81.78, C81.79, C81.90, C81.91, C81.92, C81.93, C81.95, C81.96, C81.98, C81.99, C82.00, C82.01, C82.02, C82.03, C82.05, C82.06, C82.08, C82.09, C82.10, C82.11, C82.12, C82.13, C82.14, C82.15, C82.18, C82.19, C82.20, C82.21, C82.23, C82.24, C82.28, C82.29, C82.30, C82.31, C82.35, C82.38, C82.39, C82.40, C82.41, C82.44, C82.45, C82.48, C82.50, C82.51, C82.54, C82.55, C82.56, C82.58, C82.59, C82.69, C82.80, C82.81, C82.83, C82.88, C82.89, C82.90, C82.91, C82.92, C82.93, C82.94, C82.95, C82.96, C82.97, C82.98, C82.99, C83.00, C83.01, C83.02, C83.03, C83.04, C83.05, C83.06, C83.07, C83.08, C83.09, C83.10, C83.11, C83.12, C83.13, C83.14, C83.15, C83.16, C83.17, C83.18, C83.19, C83.30, C83.31, C83.32, C83.33, C83.34, C83.35, C83.36, C83.37, C83.38, C83.39, C83.50, C83.51, C83.53, C83.55, C83.58, C83.70, C83.78, C83.79, C83.80, C83.81, C83.83, C83.84, C83.88, C83.89, C83.90, C83.91, C83.98, C83.99, C84.00, C84.04, C84.08, C84.09, C84.10, C84.18, C84.40, C84.43, C84.45, C84.48, C84.49, C84.60, C84.61, C84.63, C84.65, C84.68, C84.70, C84.71, C84.75, C84.78, C84.79, C84.90, C84.91, C84.99, C84.A0, C84.A3, C84.A5, C84.A8, C84.Z4, C84.Z9, C85.10, C85.11, C85.12, C85.13, C85.14, C85.15, C85.16, C85.17, C85.18, C85.19, C85.20, C85.22, C85.28, C85.29, C85.80, C85.81, C85.82, C85.83, C85.84, C85.85, C85.88, C85.89, C85.90, C85.91, C85.92, C85.93, C85.94, C85.95, C85.96, C85.97, C85.98, C85.99, C86.0, C86.4, C86.5, C86.6, C88.0, C88.3, C88.4, C88.8, C88.9, C90.00, C90.01, C90.02, C90.10, C90.11, C90.12, C90.20, C90.30, C90.31, C91.00, C91.01, C91.02, C91.1, C91.10, C91.11, C91.12, C91.30, C91.31, C91.32, C91.40, C91.41, C91.50, C91.51, C91.60, C91.90, C91.91, C91.92, C91.Z0, C91.Z1, C91.Z2, C92.00, C92.01, C92.02, C92.10, C92.11, C92.12, C92.22, C92.30, C92.31, C92.32, C92.40, C92.41, C92.42, C92.50, C92.51, C92.52, C92.60, C92.61, C92.62, C92.90, C92.A0, C92.A1, C92.A2, C92.Z0, C92.Z1, C92.Z2, C93.00, C93.01, C93.02, C93.10, C93.12, C93.90, C94.20, C94.31, C94.40, C94.6, C94.80, C95.00, C95.01, C95.02, C95.10, C95.11, C95.90, C95.91, C95.92, C96.2, C96.29, C96.4, C96.6, C96.9, C96.A, C96.Z, D00.00, D00.01, D00.07, D00.08, D00.1, D01.0, D01.2, D01.3, D01.49, D01.7, D02.0, D02.4, D03.0, D03.20, D03.21, D03.30, D03.39, D03.4, D03.59, D03.61, D03.62, D03.70, D03.71, D03.72, D03.8, D03.9, D04.0, D04.112, D04.20, D04.21, D04.22, D04.30, D04.39, D04.4, D04.5, D04.60, D04.61, D04.62, D04.70, D04.71, D04.72, D04.8, D04.9, D05.00, D05.01, D05.02, D05.10, D05.11, D05.12, D05.81, D05.82, D05.90, D05.91, D05.92, D06, D06.0, D06.1, D06.7, D06.9, D07.0, D07.1, D07.2, D07.30, D07.39, D07.4, D07.5, D07.61, D09.0, D09.10, D09.19, D09.3, D09.8, D09.9, D37.01, D37.02, D37.030, D37.032, D37.039, D37.05, D37.09, D37.1, D37.2, D37.3, D37.4, D37.5, D37.6, D37.8, D37.9, D38.0, D38.1, D38.2, D38.3, D38.5, D38.6, D39.0, D39.10, D39.11, D39.12, D39.8, D39.9, D40.0, D41.00, D41.01, D41.02, D41.10, D41.20, D41.22, D41.4, D41.8, D41.9, D42.0, D42.1, D42.9, D43.0, D43.1, D43.2, D43.4, D43.9, D44.0, D44.10, D44.11, D44.12, D44.3, D44.4, D44.6, D44.7, D44.9, D45, D46.0, D46.1, D46.20, D46.21, D46.22, D46.4, D46.9, D46.A, D46.B, D46.C, D46.Z, D47.01, D47.02, D47.09, D47.1, D47.2, D47.3, D47.4, D47.9, D47.Z, D47.Z1, D47.Z2, D47.Z9, D48.0, D48.1, D48.2, D48.3, D48.4, D48.5, D48.60, D48.61, D48.62, D48.7, D48.9, D49.0, D49.1, D49.2, D49.3, D49.4, D49.5, D49.511, D49.512, D49.519, D49.59, D49.6, D49.7, D49.81, D49.89, D49.9 |
| Cardiovascular  Disease (CVD) | 410.00, 410.01, 410.02, 410.1, 410.10, 410.11, 410.12, 410.20, 410.21, 410.22, 410.31, 410.32, 410.40, 410.41, 410.42, 410.50, 410.51, 410.61, 410.70, 410.71, 410.72, 410.80, 410.81, 410.82, 410.9, 410.90, 410.91, 410.92, 411.0, 411.1, 411.81, 411.89, 412, 413.0, 413.1, 413.9, 414.0, 414.00, 414.01, 414.02, 414.03, 414.04, 414.05, 414.06, 414.07, 414.10, 414.11, 414.12, 414.19, 414.2, 414.3, 414.4, 414.8, 414.9, 415.0, 415.1, 415.11, 415.12, 415.13, 415.19, 416, 416.0, 416.1, 416.2, 416.8, 416.9, 417.0, 417.1, 417.8, 417.9, 420, 420.0, 420.90, 420.91, 420.99, 421.0, 421.1, 421.9, 422.0, 422.90, 422.91, 422.93, 422.99, 423.0, 423.1, 423.2, 423.3, 423.8, 423.9, 424.0, 424.1, 424.2, 424.3, 424.90, 424.91, 425, 425.0, 425.1, 425.11, 425.18, 425.2, 425.3, 425.4, 425.5, 425.7, 425.8, 425.9, 426.0, 426.10, 426.11, 426.12, 426.13, 426.2, 426.3, 426.4, 426.50, 426.51, 426.52, 426.53, 426.54, 426.6, 426.7, 426.82, 426.89, 426.9, 427.0, 427.1, 427.2, 427.31, 427.32, 427.41, 427.42, 427.5, 427.60, 427.61, 427.69, 427.81, 427.89, 427.9, 428, 428.0, 428.1, 428.2, 428.20, 428.21, 428.22, 428.23, 428.3, 428.30, 428.31, 428.32, 428.33, 428.40, 428.41, 428.42, 428.43, 428.9, 429.0, 429.1, 429.2, 429.3, 429.4, 429.5, 429.6, 429.71, 429.79, 429.81, 429.82, 429.83, 429.89, 429.9, I20.9, I21.09, I21.3, I25.10, I25.2, I25.84, I25.9, I48.91, I50.9, I63.9, I65.23, I65.29, I67.2, I67.9, I73.9 |
| Chronic Liver Disease (CLD) | 571.8, 571.9, 572.8, K71.3, K71.51 |
| Chronic Obstructive Pulmonary Disease (COPD) | 491.2, 496, J44.0, J44.1, J44.9 |
| Diabetes | 250, 250.00, 250.01, 250.02, 250.03, 250.10, 250.11, 250.12, 250.13, 250.20, 250.21, 250.22, 250.3, 250.30, 250.31, 250.32, 250.33, 250.40, 250.41, 250.42, 250.43, 250.50, 250.51, 250.52, 250.53, 250.6, 250.60, 250.61, 250.62, 250.63, 250.70, 250.71, 250.72, 250.73, 250.80, 250.81, 250.82, 250.83, 250.90, 250.91, 250.92, 250.93, E10.10, E10.11, E10.21, E10.22, E10.29, E10.311, E10.319, E10.3213, E10.3219, E10.329, E10.3291, E10.3292, E10.3293, E10.3299, E10.331, E10.3311, E10.3312, E10.3313, E10.3319, E10.339, E10.3391, E10.3393, E10.3399, E10.3413, E10.3491, E10.3493, E10.3499, E10.351, E10.3511, E10.3512, E10.3513, E10.3519, E10.3521, E10.3522, E10.3523, E10.3531, E10.3532, E10.3533, E10.3539, E10.3542, E10.3543, E10.3551, E10.3553, E10.3559, E10.359, E10.3591, E10.3592, E10.3593, E10.3599, E10.36, E10.39, E10.40, E10.41, E10.42, E10.43, E10.44, E10.49, E10.51, E10.52, E10.59, E10.610, E10.618, E10.620, E10.621, E10.622, E10.628, E10.641, E10.649, E10.65, E10.69, E10.8, E10.9, E11, E11.00, E11.01, E11.10, E11.11, E11.21, E11.22, E11.29, E11.311, E11.319, E11.321, E11.3211, E11.3212, E11.3213, E11.3219, E11.329, E11.3291, E11.3292, E11.3293, E11.3299, E11.331, E11.3311, E11.3312, E11.3313, E11.3319, E11.339, E11.3391, E11.3392, E11.3393, E11.3399, E11.3411, E11.3412, E11.3413, E11.3419, E11.349, E11.3491, E11.3492, E11.3493, E11.3499, E11.351, E11.3511, E11.3512, E11.3513, E11.3519, E11.3521, E11.3522, E11.3531, E11.3532, E11.3533, E11.3541, E11.3542, E11.3543, E11.3551, E11.3552, E11.3553, E11.3559, E11.359, E11.3591, E11.3592, E11.3593, E11.3599, E11.36, E11.37X1, E11.37X9, E11.39, E11.40, E11.41, E11.42, E11.43, E11.44, E11.49, E11.51, E11.52, E11.59, E11.610, E11.618, E11.620, E11.621, E11.622, E11.628, E11.630, E11.638, E11.641, E11.649, E11.65, E11.69, E11.8, E11.9, E13.00, E13.01, E13.10, E13.21, E13.22, E13.29, E13.311, E13.319, E13.3293, E13.3299, E13.3391, E13.3499, E13.3542, E13.3543, E13.3593, E13.3599, E13.39, E13.40, E13.41, E13.42, E13.43, E13.49, E13.51, E13.52, E13.59, E13.610, E13.620, E13.621, E13.622, E13.628, E13.649, E13.65, E13.69, E13.8, E13.9 |
| Human Immunodeficiency virus (HIV) infection | 042, 079.53, 795.71, B20, R75, V08, Z11.4, Z20.6, Z21, Z71.7, Z83.0, |
| Hypertension | 401, 401.0, 401.1, 401.9, 402.00, 402.01, 402.10, 402.11, 402.90, 402.91, 403, 403.0, 403.00, 403.01, 403.10, 403.11, 403.90, 403.91, 404.00, 404.01, 404.02, 404.03, 404.10, 404.11, 404.12, 404.13, 404.9, 404.90, 404.91, 404.92, 404.93, 405.01, 405.09, 405.11, 405.19, 405.91, 405.99, I10, I11.0, I11.9, I12.0, I12.9, I13.0, I13.10, I13.11, I13.2, I15.0, I15.1, I15.2, I15.8, I15.9, I16.0, I16.1, I16.9 |
| Immunological disease or disorder | 340, 357.0, 357.81, 358.00, 358.01, 437.4, 446.0, 446.21, 446.4, 446.5, 446.7, 447.6, 695.4, 696.0, 696.1, 696.2, 696.8, 710.0, 710.2, 714.0, 714.1, 714.2, 714.31, 714.33, 714.4, 714.81, 714.89, 714.9, 775.2, G35, G61.0, G61.81, G70.00, G70.01, I67.7, I77.6, I79.1, L40.0, L40.1, L40.4, L40.50, L40.52, L40.59, L40.8, L40.9, L41.9, L93.0, L93.2, M08.3, M30.0, M31.0, M31.30, M31.31, M31.4, M31.5, M31.6, M31.7, M32.10, M32.12, M32.13, M32.14, M32.19, M32.8, M32.9, M35.00, M35.01, M35.02, M35.03, M35.04, M35.09 |
| Transplant | Z94.84 |

- 1. Immune modulator medications

Immune modulator medications under this category include alemtuzumab, anti-thymocyte globulin, azathioprine, basiliximab, belatacept, cyclosporine, everolimus, leflunomide, methotrexate, mycophenolate mofetil, mycophenolate sodium, mycophenolic acid, rituximab, sirolimus, and tacrolimus.

- 1. Vaccine status was classified as follows

SARS-CoV-2 Vaccination status was based on status on admission. We used four categories:

- None – no COVID-19 vaccine dose
- Incompete – only one dose of a COVID-19 vaccine except for the Ad26.COV2.S (Johnson & Johnson) vaccine
- Full – one dose of the Ad26.COV2.S (Johnson & Johnson) vaccine or two doses of any other COVID-19 vaccine
- Boosted – two or more doses with the first dose being Ad26.COV2.S (Johnson & Johnson) OR three or more doses of any other COVID-19 vaccine

1. **Missing data imputation**

Missing data was imputed using single imputation with missForest (1). missForest uses gradient boosted trees to impute. Following imputation guidelines, imputation models included outcomes, exposures, covariates, and additional variables that may contribute information.

For the antecedent use analysis, predictors include: statin use, site, month of admission, age, gender, race, Hispanic ethnicity, insurance status (binary), county of residence, body mass index (BMI), ever smoking, comorbidities (binary: asthma, hypertension (HTN), diabetes mellitus (DM), cardiovascular disease (CVD), chronic obstructive pulmonary disease (COPD), renal disease, chronic liver disease (CLD), cancer, HIV, and immune disorders), outcome variable, length of stay, and clinical variables (heart rate, respiratory rate, systolic and diastolic blood pressure (SBP, DBP), and oxygen saturation).

Sample code:

###load library

library(missForest)

###load wide data set for imputation (fin_data is a wide dataset with outcomes and covariates)

mi.data <- fin_data %>% filter(include == 1) %>%

dplyr::select(ir_id, statin_4cat, site_num, month_adm, age, sex, race, hispanic, ins_yn, county_grp, bmi0, ever_smoke_yn, asthma_bl, htn_bl, dm_bl, cvd_bl, copd_bl, renal_bl, cld_bl, cancer_bl, hiv_bl, imm_bl, outcome_yn, outcome_inpt_yn, icu_yn, intub_yn, los, death_yn, death_inpt_yn, hr0, rr0, sbp0, dbp0, spo20) %>%

distinct(ir_id, .keep_all=TRUE) %>%

dplyr::select(-ir_id, -race_4cat, -race,-hispanic) %>%

mutate(site_num = factor(site_num), statin_4cat = factor(statin_4cat), county_grp = relevel(factor(county_grp), ref="cook"))

####set up parallelization and impute

doParallel::registerDoParallel(cores = detectCores()-1) # set based on number of CPU cores

doRNG::registerDoRNG(seed = 1234)

mf.obj <- missForest(mi.data, verbose = TRUE, parallelize = 'forests', mtry=9, maxiter=20)

1. **Augmented Inverse Propensity Weighting with Targeted Maximum Likelihood Estimation**

Augmented inverse propensity weighting (AIPW) was combined with targeted maximum likelihood estimation (TMLE) used to estimate the risk difference for the four inpatient outcomes (composite, intensive care unit (ICU) admission, intubation, inpatient death) between antecedent statin users and non-users. This involved first estimating the TMLE object followed with using the AIPW to use the estimated TMLE function to calculate the risk differences (2,3).

TMLE was implemented using the tmle and superlearner packages (3,4). It involves specifying the outcome, exposure, covariates, and the superlearner libraries to be used for estimating the treatment and outcome models. For all analyses, we used the following libraries: SL.mean, SL.glm, SL.ranger (random forest), SL.glmnet (lasso by default), and SL.xgboost (booster trees). Covariates varied depending on the exposure of interest.

Covariates when the exposure was antecedent use included the following sets of variables:

- Baseline: age, male gender, Hispanic ethnicity, race category, insurance status, overweight (BMI>=25), ever smoking, SARS-CoV-2 vaccination status, county, comorbidity (HTN, DM, CVD, CLD, COPD, Asthma), and month of admission
- Site variables: site of admission
- Clinical variables: HR, RR, SBP, DBP, oxygen saturation on admission

Sample code for the composite binary outcome and antecedent use as an exposure is as follows:

###load library

library(tmle)

library(superlearner)

library(parallel)

###set up variables (mod.data2 is the clean wide dataset)

outcome <- mod.data2$outcome_inpt_yn

exposure <- mod.data2$statin_ant_yn

wvars <- bl_covariates ###bl_covariates is a vector containing variable names of covariates

w_df <- mod.data2[,wvars] #we use the same covariates for both treatment and outcome models

###set-up parallelization

cluster = parallel::makeCluster(detectCores()-2)

parallel::clusterSetRNGStream(cluster, iseed=12345)

options(mc.cores=detectCores()-2)

###set-up learners: g.lib are learners for the treatment model and Q.lib is for the outcome model

g.lib = c("SL.mean","SL.glm", "SL.glmnet",

"SL.ranger", "SL.xgboost")

Q.lib = g.lib

###run TMLE

tmle_comp <- tmle(Y = outcome, A = exposure,W = w_df,

Q.SL.library=Q.lib,

g.SL.library=g.lib,

family="binomial",

cvQinit=TRUE, V=10)

###run AIPW with fitted tmle object

outcomevar = outcome

AIPW_tmle$new(A=exposure, Y=outcomevar, tmle_fit = comp, verbose = TRUE)$summary()

1. **TMLE for three-category exposures**

For sensitivity analysis using a categorical statin exposure (none, low-intensity, high-intensity), we used TMLE as implemented in the tmle3 package (5). This was used since the older tmle package can only accommodate binary exposures. The covariates included were as described previously. The only notable change (aside from the syntax) is the use of an independent binomial regression learner instead of a generalized linear model learner for estimating the three-category exposure model. We initially used multinomial regression, but it was producing errors. The estimates were still risk differences, but we used non-user as the reference group. Sample code is as follows:

###load libraries

library(tmle3)

library(sl3)

###set-up nodes

node_list <- list(

W = bl_covariates, #vector with variable names of covariates

A = "statin_int", #three category exposure

Y = "outcome_inpt_yn" #outcome

)

###define ate_spec

ate_spec <- tmle_ATE(

treatment_level = "high-intensity",

control_level = "non-user"

)

#define tmle task

tmle_task <- ate_spec$make_tmle_task(hi.mod.data, node_list)

###set-up learners

lrnr_mean <- make_learner(Lrnr_mean)

lrnr_glm <- make_learner(Lrnr_glm)

lrnr_rf <- make_learner(Lrnr_ranger)

lrnr_lasso <- make_learner(Lrnr_glmnet, alpha=1) #alpha=1 to run lasso regression

lrnr_xgb <- make_learner(Lrnr_xgboost)

lrnr_nnet <- make_learner(Lrnr_nnet) #for 3 category A

### define metalearners appropriate to data types

ls_metalearner <- make_learner(Lrnr_nnls)

mn_metalearner <- make_learner(

Lrnr_solnp, metalearner_linear_multinomial,

loss_loglik_multinomial

)

Y_lrn_list <- list(lrnr_mean, lrnr_rf, lrnr_glm, lrnr_lasso, lrnr_xgb)

A_lrn_list <- list(lrnr_mean, lrnr_rf, lrnr_nnet, lrnr_lasso, lrnr_xgb) #use nnet instead of glm

sl_Y <- Lrnr_sl$new(

learners = Y_lrn_list,

metalearner = ls_metalearner

)

sl_A <- Lrnr_sl$new(

learners = A_lrn_list,

metalearner = mn_metalearner

)

learner_list <- list(A = sl_A, Y = sl_Y)

###initial likelihood

initial_likelihood <- ate_spec$make_initial_likelihood(

tmle_task,

learner_list

)

###write other parameters

tsm_spec <- tmle_TSM_all()

targeted_likelihood <- Targeted_Likelihood$new(initial_likelihood)

all_tsm_params <- tsm_spec$make_params(tmle_task, targeted_likelihood)

###use delta method for custom ATE

ate_param <- define_param(

Param_delta, targeted_likelihood,

delta_param_ATE,

list(all_tsm_params[[3]], all_tsm_params[[2]])

)

ate_param2 <- define_param(

Param_delta, targeted_likelihood,

delta_param_ATE,

list(all_tsm_params[[3]], all_tsm_params[[1]])

)

ate_param3 <- define_param(

Param_delta, targeted_likelihood,

delta_param_ATE,

list(all_tsm_params[[2]], all_tsm_params[[1]])

)

###combine all parameters for estimation

all_params <- c(all_tsm_params,

ate_param, ate_param2, ate_param3)

###fit tmle

tmle_fit_multiparam <- fit_tmle3(

tmle_task, targeted_likelihood, all_params,

targeted_likelihood$updater

)

###show results

tmle_fit_multiparam$tmle_task

1. **Sensitivity analyses for antecedent statin use using inverse propensity scores**

For this analysis, we used inverse propensity weighting where we only specified a treatment model and then used the weights to estimate the risk difference (or odds ratio) with a weighted outcome model with only antecedent use in the right-hand side of the equation. IPW were estimated using the WeightIt package with two approaches: logistic regression (glm) or boosted trees (gbm). Since, we used weights, the outcome model was estimated using a robust variance estimator as implemented in the survey package. Covariates for the treatment model were the same as the main analyses. Truncation of weights greater than 10 were done. Sample code is as follows:

###load libraries

library(survey)

library(emmeans)

library(WeightIt)

###set-up equation for treatment model

bl.form <- formula(statin_ant_yn ~ age_rcs1 + age_rcs2 + age_rcs3 + age_rcs4 +

male_gender + hispanic +

race_5cat_asian + race_5cat_black + race_5cat_multiracial + race_5cat_other +

ins_yn + overweight + ever_smoke_yn +

vacc_stat_adm_inc + vacc_stat_adm_full + vacc_stat_adm_boosted +

county_grp_dekalb + county_grp_dupage + county_grp_kane +

county_grp_lake + county_grp_mchenry + county_grp_others +

county_grp_non_il + htn_bl + dm_bl + cvd_bl + cld_bl + copd_bl + asthma_bl +

cancer_bl + imm_bl + hiv_bl + renal_bl +

month_adm_rcs1 + month_adm_rcs2 + month_adm_rcs3 + month_adm_rcs4)

###calculate weights

w.bl <- weightit(bl.form,

data = mod.data2, estimand = "ATE", method = "ps", stabilize = TRUE)

wts <- weightit_object$weights

wts[wts>=10] <- 10 #truncate high weights at 10

###run outcome model

d.w <- svydesign(~1, weights = wts, family='binomial', data = mod.data2)

mod.comp <- svyglm(outcome_inpt_yn ~ statin_ant_yn, design = d.w)

###get odds ratio

tidy(mod.comp, exp=TRUE, conf.int=TRUE) %>% filter(term=="statin_ant_yn")

###get risk difference

em.comp <- emmeans(mod.comp, specs=~statin_ant_yn, type='response')

pairs(em.comp, reverse=TRUE)

1. **References cited**

1. Stekhoven DJ, Bühlmann P. Missforest-Non-parametric missing value imputation for mixed-type data. Bioinformatics. 2012;28(1):112–8.

2. Zhong Y, Brooks MM, Kennedy EH, Bodnar LM, Naimi AI. Use of Machine Learning to Estimate the Per-Protocol Effect of Low-Dose Aspirin on Pregnancy Outcomes: A Secondary Analysis of a Randomized Clinical Trial. JAMA Netw Open. 2022 Mar 9;5(3):e2143414.

3. Gruber S, Laan MJ van der. **tmle** : An *R* Package for Targeted Maximum Likelihood Estimation. J Stat Soft [Internet]. 2012 [cited 2022 Dec 7];51(13). Available from: http://www.jstatsoft.org/v51/i13/

4. Naimi AI, Balzer LB. Stacked generalization: an introduction to super learning. Eur J Epidemiol. 2018 May;33(5):459–64.

5. Coyle J. {tmle3}: The Extensible {TMLE} Framework [Internet]. 2022. Available from: https://github.com/tlverse/tmle3
