# Supplementary material for: Association of statin use with outcomes of patients admitted with COVID-19: an analysis of electronic health records using superlearner
Source: BMC Infect Dis. 2023 Feb 24;23:115. doi: 10.1186/s12879-023-08026-0 (PMC9951166; doi:10.1186/s12879-023-08026-0)
Supplement: Supplementary file 2 — Additional file 2: Table S1. Demographic and Clinical Characteristics of Included versus Excluded Admissions, Northwestern Medical Group, March 2020-September 2022. Table S2. Sensitivity Analysis for Risk Differences of Inpatient Outcomes using Outcome Regression and Inverse Propensity Weighting, Northwestern Medical Group, March 2020-September 2022. Table S3. Sensitivity Analysis for Antecedent Statin and Risk Differences of Inpatient Outcomes Comparing only Never users to Antecedent users, Northwestern Medical Group, March 2020-September 2022. [file 12879_2023_8026_MOESM2_ESM.docx]

**Additional File 2. Supplemental Tables**

Association of Statin Use with Outcomes of Patients Admitted with COVID-19: An Analysis of Electronic Health Records using Superlearner

Adovich S. Rivera MD*^1,2^, Omar Al-Heeti MD*^3^, Lucia C. Petito PhD^4^, Mathew J. Feinstein MD MS^4,6^, Chad J. Achenbach MD, MPH^3,4,5^, Janna Williams MD^3^, Babafemi Taiwo MBBS^3,5^

^1^Institute for Public Health and Management, Feinberg School of Medicine, Chicago, IL, 60611

2Department of Research and Evaluation, Kaiser Permanente Southern California, Pasadena, CA, 91101

^3^Department of Medicine, Division of Infectious Diseases, Northwestern University Feinberg School of Medicine, Chicago, IL 60611

^4^Department of Preventive Medicine, Division of Biostatistics, Feinberg School of Medicine, Chicago, IL, 60611

^5^Havey Institute for Global Health, Northwestern University Feinberg School of Medicine, Chicago, IL 606011

^6^Department of Medicine, Division of Cardiology, Feinberg School of Medicine, Chicago, IL, 60611

^*co-first author^

**Table S1. Demographic and Clinical Characteristics of Included versus Excluded Admissions, Northwestern Medical Group, March 2020-September 2022**

|  | Excluded  (n=3,818) | Included  (n=15,524) |
| --- | --- | --- |
| **Baseline variables** |  |  |
| Age (mean (SD)) | 30.85 (5.64) | 67.42 (14.32) |
| Male sex (%) | 1170 (30.6) | 8021 (51.7) |
| Race (%) |  |  |
| Asian | 155 ( 4.2) | 465 ( 3.0) |
| Black | 704 (19.0) | 2074 (13.6) |
| Multiracial^1^ | 70 ( 1.9) | 251 ( 1.6) |
| Other^2^ | 653 (17.6) | 1365 ( 9.0) |
| White | 2127 (57.3) | 11096 (72.8) |
| Hispanic/Latino/x (%) | 1151 (30.1) | 2294 (14.8) |
| County (%) |  |  |
| Cook | 1565 (42.4) | 4260 (28.4) |
| DeKalb | 232 ( 6.3) | 1179 ( 7.8) |
| DuPage | 508 (13.7) | 2557 (17.0) |
| Kane | 303 ( 8.2) | 1552 (10.3) |
| Lake | 492 (13.3) | 2111 (14.1) |
| McHenry | 330 ( 8.9) | 2373 (15.8) |
| Non-Illinois | 121 ( 3.3) | 468 ( 3.1) |
| Others^3^ | 144 ( 3.9) | 521 ( 3.5) |
| Site |  |  |
| 1 | 665 (17.4) | 3047 (19.6) |
| 2 | 227 ( 5.9) | 1418 ( 9.1) |
| 3 | 246 ( 6.4) | 1548 (10.0) |
| 4 | 221 ( 5.8) | 1089 ( 7.0) |
| 5 | 457 (12.0) | 1923 (12.4) |
| 6 | 190 ( 5.0) | 1826 (11.8) |
| 7 | 1747 (45.8) | 4423 (28.5) |
| 8 | 41 ( 1.1) | 166 ( 1.1) |
| 9 | 19 ( 0.5) | 13 ( 0.1) |
| 10 | 5 ( 0.1) | 71 ( 0.5) |
| BMI ≥ 25 (%) | 2652 (80.1) | 10244 (73.4) |
| Ever smoked (%) | 650 (17.0) | 2973 (19.2) |
| Vaccination status on admission (%) |  |  |
| None | 2884 (75.5) | 11009 (70.9) |
| Incomplete | 522 (13.7) | 2157 (13.9) |
| Full | 144 ( 3.8) | 384 ( 2.5) |
| Boosted | 268 ( 7.0) | 1974 (12.7) |
| Quarter and Year of Admission (%) |  |  |
| 2020 Quarter 1 | 49 ( 1.3) | 259 ( 1.7) |
| 2020 Quarter 2 | 373 ( 9.8) | 1599 (10.3) |
| 2020 Quarter 3 | 231 ( 6.1) | 619 ( 4.0) |
| 2020 Quarter 4 | 535 (14.0) | 3110 (20.0) |
| 2021 Quarter 1 | 315 ( 8.3) | 1389 ( 8.9) |
| 2021 Quarter 2 | 292 ( 7.6) | 817 ( 5.3) |
| 2021 Quarter 3 | 281 ( 7.4) | 840 ( 5.4) |
| 2021 Quarter 4 | 429 (11.2) | 1771 (11.4) |
| 2022 Quarter 1 | 615 (16.1) | 2080 (13.4) |
| 2022 Quarter 2 | 320 ( 8.4) | 1182 ( 7.6) |
| 2022 Quarter 3 | 317 ( 8.3) | 1471 ( 9.5) |
| 2022 Quarter 4 | 61 ( 1.6) | 387 ( 2.5) |
| **Initial Clinical Presentation** |  |  |
| Heart Rate (bpm) | 94.26 (19.36) | 89.31 (19.34) |
| Respiratory rate (bpm) | 19.43 (5.13) | 20.97 (5.60) |
| Systolic blood pressure (mmHg) | 127.29 (18.49) | 136.65 (25.66) |
| Diastolic blood pressure (mmHg) | 76.24 (13.02) | 74.49 (14.54) |
| Oxygen saturation (%) | 96.89 (4.89) | 94.69 (5.86) |
| **Comorbidities at baseline (%)** |  |  |
| Asthma | 610 (16.0) | 2221 (14.3) |
| Cancer | 332 ( 8.7) | 4920 (31.7) |
| Cardiovascular disease | 495 (13.0) | 8689 (56.0) |
| Chronic liver disease | 95 ( 2.5) | 692 ( 4.5) |
| COPD | 16 ( 0.4) | 2200 (14.2) |
| Cardiovascular disease | 495 (13.0) | 8689 (56.0) |
| Diabetes mellitus | 432 (11.3) | 5753 (37.1) |
| HIV | 163 ( 4.3) | 456 ( 2.9) |
| Hypertension | 627 (16.4) | 10915 (70.3) |
| Immune disorder | 234 ( 6.1) | 1779 (11.5) |
| Renal disease | 385 (10.1) | 5640 (36.3) |
| **Treatment during admission (%)** |  |  |
| Dexamethasone | 1141 (29.9) | 7488 (48.2) |
| Remdesivir | 651 (17.1) | 6444 (41.5) |
| Sarilumab | 10 ( 0.3) | 16 ( 0.1) |
| Tocilizumab | 60 ( 1.6) | 608 ( 3.9) |
| Bamlanivumab | 0 ( 0.0) | 22 ( 0.1) |
| Steroids | 1376 (36.0) | 8753 (56.4) |
| Immune modulator^4^ | 123 ( 3.2) | 686 ( 4.4) |
| **Outcomes** |  |  |
| Composite inpatient outcome (inpatient death, ICU, mech vent) (%) | 333 ( 8.7) | 2793 (18.0) |
| ICU admission (%) | 331 ( 8.7) | 2648 (17.1) |
| Intubation (%) | 117 ( 3.1) | 1145 ( 7.4) |
| Inpatient death (%) | 26 ( 0.7) | 779 ( 5.0) |
| Days from admission to comp outcome (median [IQR]) | 0.22 [0.13, 0.93] | 0.33 [0.15, 2.86] |
| Length of Stay (median [IQR]) | 2.85 [2.06, 4.59] | 4.80 [2.80, 8.39] |

Notes: BMI – body mass index, bpm – beats or breathes per minute, COPD – chronic obstructive pulmonary disease, ICU – intensive care unit. ^1^ – Multiracial individuals are people who report more than two categories (e.g., Asian and Black), ^2^ – Others include American Indian, Alaska Native, Native Hawaiian, Pacific-Islander, Guamanian, and Chamorro or chose other or none of the above. ^3^ – Other Illinois counties include all other counties not listed in the table. ^4^ – See methods supplement for complete list of immune-modulator drugs. Variables with missing data: Body mass index (11%), county (3%), race (2%), insurance (0.1%), Systolic blood pressure (0.04%), Diastolic blood pressure (0.04%), Oxygen saturation (0.02%).

**Table S2. Sensitivity Analysis for Risk Differences of Inpatient Outcomes using Outcome Regression and Inverse Propensity Weighting, Northwestern Medical Group, March 2020-September 2022**

1. **Risk Differences adjusting for baseline covariates only (95% CI)**

| Outcome | Unweighted | Weighted (glm) | Weighted (gbm) |
| --- | --- | --- | --- |
| Composite outcome | -3.8 (-5.2 to -2.4)* | -3.4 (-5.2 to -1.6)* | -3.1 (-4.9 to -1.4)* |
| ICU | -3.7 (-5.1 to -2.4)* | -3.4 (-5.2 to -1.6)* | -3.0 (-4.7 to -1.3)* |
| Intubation | -1.6 (-2.3 to -0.8)* | -1.9 (-3.2 to -0.6)* | -1.7 (-2.9 to -0.6)* |
| Inpatient death | -0.3 (-0.9 to 0.2) | -0.6 (-1.5 to 0.4) | -0.6 (-1.5 to 0.3) |

1. **Risk Differences adjusting for baseline and site variables (95% CI)**

| Outcome | Unweighted | Weighted (glm) | Weighted (gbm) |
| --- | --- | --- | --- |
| Composite outcome | -3.8 (-5.1 to -2.4)* | -3.5 (-5.3 to -1.7)* | -3.1 (-4.9 to -1.3)* |
| ICU | -3.6 (-4.9 to -2.3)* | -3.4 (-5.2 to -1.6)* | -3.0 (-4.7 to -1.3)* |
| Intubation | -1.5 (-2.2 to -0.8)* | -1.9 (-3.2 to -0.6)* | -1.7 (-2.9 to -0.5)* |
| Inpatient death | -0.3 (-0.9 to 0.2) | -0.6 (-1.5 to 0.4) | -0.5 (-1.4 to 0.4) |

1. **Risk Differences adjusting for baseline, site, and clinical variables (95% CI)**

| Outcome | Unweighted | Weighted (glm) | Weighted (gbm) |
| --- | --- | --- | --- |
| Composite outcome | -3.4 (-4.7 to -2.1)* | -3.2 (-5 to -1.4)* | -2.9 (-4.7 to -1.2)* |
| ICU | -3.2 (-4.5 to -2.0)* | -3.2 (-5 to -1.4)* | -2.9 (-4.6 to -1.2)* |
| Intubation | -1.2 (-1.8 to -0.5)* | -1.7 (-3.0 to -0.4)* | -1.8 (-3.0 to -0.7)* |
| Inpatient death | -0.3 (-0.7 to 0.2) | -0.5 (-1.4 to 0.5) | -0.5 (-1.4 to 0.3) |

**Notes:** CI – confidence interval * - 95% CI lies on the same side of null (zero) suggesting significant difference between the two groups. Unweighted analysis corresponds to outcome regression or the usual multivariable regression for the outcome. Weighted analysis estimates weights using a treatment model adjusting for covariates. The outcome model is a weighted regression adjusting only for treatment status. ‘glm’ refers to weighting using logistic regression while ‘gbm’ uses gradient boosted models. CI for weighted models were calculated using robust variance estimators. Adjustment variables are the same as the models for the main analysis.

**Table S3. Sensitivity Analysis for Antecedent Statin and Risk Differences of Inpatient Outcomes Comparing only Never users to Antecedent users, Northwestern Medical Group, March 2020-September 2022**

| **Outcome** | **Risk Difference (95% CI)** | | |
| --- | --- | --- | --- |
|  | **Baseline** | **Baseline + Site** | **Baseline + Site + Clinical** |
| Composite outcome | -2.0  (-3.5, -0.5)* | -2.0  (-3.5, -0.5)* | -0.8  (-1.9, 0.3)* |
| ICU | -1.3  (-2.3, -0.4)* | -1.2  (-2.1, -0.3)* | -0.7  (-1.4, 0.03) |
| Intubation | -2.1  (-3.7, -0.5)* | -1.9  (-3.2, -0.7)* | -0.9  (-2.0, 0.2) |
| Inpatient death | -0.5  (-1.2, 0.2) | -0.5  (-1.2, 0.2) | -0.1  (-0.7, 0.4) |

Notes: CI – confidence interval * - 95% CI lies on the same side of null (zero) suggesting significant difference between the two groups. Never users are those who never start statins, so they exclude people who start statin just before (<30 days) admission and those who initiate during admission. Adjustment variables are the same as the models for the main analysis.
