# Supplementary material for: Association of statin use with outcomes of patients admitted with COVID-19: an analysis of electronic health records using superlearner
Source: BMC Infect Dis. 2023 Feb 24;23:115. doi: 10.1186/s12879-023-08026-0 (PMC9951166; doi:10.1186/s12879-023-08026-0)
Supplement: Supplementary file 3 — Additional file 3: Figure S1. Love plots for covariate balance using different weighting methods with baseline variables, Northwestern Medical Group, March 2020-September 2022. Figure S2. Love plots for covariate balance using different weighting methods with baseline and site variables, Northwestern Medical Group, March 2020-September 2022. Figure S3. Love plots for covariate balance using different weighting methods with baseline, site and clinical variables, Northwestern Medical Group, March 2020-September 2022. [file 12879_2023_8026_MOESM3_ESM.docx]

**Additional File 3. Supplemental Figures**

Association of Statin Use with Outcomes of Patients Admitted with COVID-19: An Analysis of Electronic Health Records using Superlearner

Adovich S. Rivera MD*^1,2^, Omar Al-Heeti MD*^3^, Lucia C. Petito PhD^4^, Mathew J. Feinstein MD MS^4,6^, Chad J. Achenbach MD, MPH^3,4,5^, Janna Williams MD^3^, Babafemi Taiwo MBBS^3,5^

^1^Institute for Public Health and Management, Feinberg School of Medicine, Chicago, IL, 60611

2Department of Research and Evaluation, Kaiser Permanente Southern California, Pasadena, CA, 91101

^3^Department of Medicine, Division of Infectious Diseases, Northwestern University Feinberg School of Medicine, Chicago, IL 60611

^4^Department of Preventive Medicine, Division of Biostatistics, Feinberg School of Medicine, Chicago, IL, 60611

^5^Havey Institute for Global Health, Northwestern University Feinberg School of Medicine, Chicago, IL 606011

^6^Department of Medicine, Division of Cardiology, Feinberg School of Medicine, Chicago, IL, 60611

^*co-first author^

**List of Supplemental Figures**

Figure S1. Love plots for covariate balance using different weighting methods with baseline variables, Northwestern Medical Group, March 2020-September 2022.

Figure S2. Love plots for covariate balance using different weighting methods with baseline and site variables, Northwestern Medical Group, March 2020-September 2022.

Figure S3. Love plots for covariate balance using different weighting methods with baseline, site and clinical variables, Northwestern Medical Group, March 2020-September 2022.

**Figure S1. Love plots for covariate balance using different weighting methods with baseline variables, Northwestern Medical Group, March 2020-September 2022.**


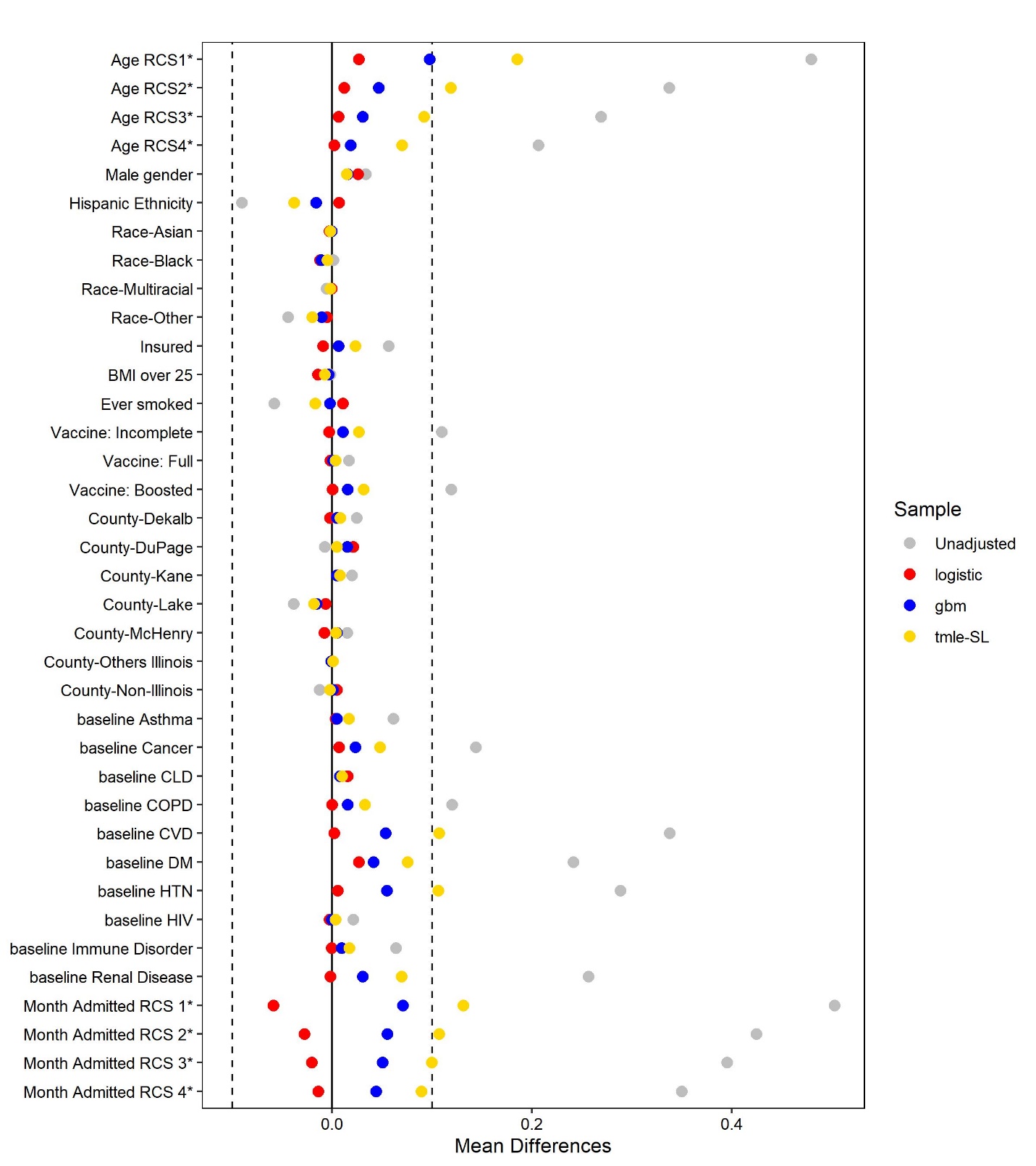


Note: * - standardized mean differences. Unadjusted – no weighting applied to calculated differences. logistic – used weights derived from a logistic regression model. gbm – weights derived from the a gradient boosted model. tmle-SL – weights obtained from the targeted maximum likelihood estimation object with super learner. BMI – body mass index, CLD – chronic liver disease, COPD – chronic obstructive pulmonary disease, CVD – cardiovascular disease, DM – diabetes mellitus, HTN – hypertension, HIV – human immunodeficiency virus, RCS – restricted cubic spline. Multiracial individuals are people who report more than two categories (e.g., Asian and Black), Other races include American Indian, Alaska Native, Native Hawaiian, Pacific-Islander, Guamanian, and Chamorro or chose other or none of the above. Other Illinois counties include all other counties not listed in the figure.

**Figure S2. Love plots for covariate balance using different weighting methods with baseline and site variables, Northwestern Medical Group, March 2020-September 2022.**


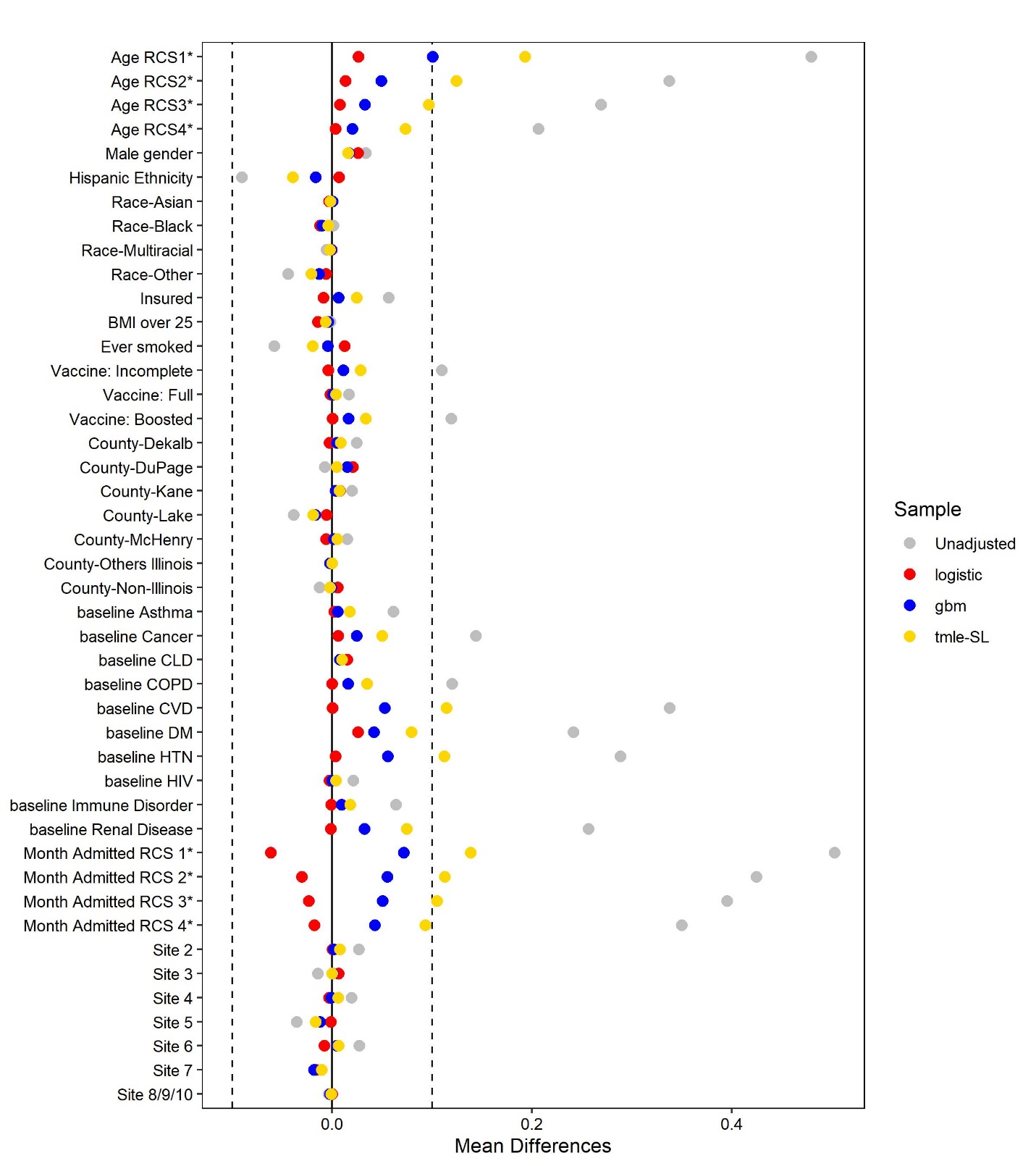
 Note: * - standardized mean differences. Unadjusted – no weighting applied to calculated differences. logistic – used weights derived from a logistic regression model. gbm – weights derived from the a gradient boosted model. tmle-SL – weights obtained from the targeted maximum likelihood estimation object with super learner. BMI – body mass index, CLD – chronic liver disease, COPD – chronic obstructive pulmonary disease, CVD – cardiovascular disease, DM – diabetes mellitus, HTN – hypertension, HIV – human immunodeficiency virus, RCS – restricted cubic spline. Multiracial individuals are people who report more than two categories (e.g., Asian and Black), Other races include American Indian, Alaska Native, Native Hawaiian, Pacific-Islander, Guamanian, and Chamorro or chose other or none of the above. Other Illinois counties include all other counties not listed in the figure.

**Figure S3. Love plots for covariate balance using different weighting methods with baseline, site and clinical variables, Northwestern Medical Group, March 2020-September 2022.**


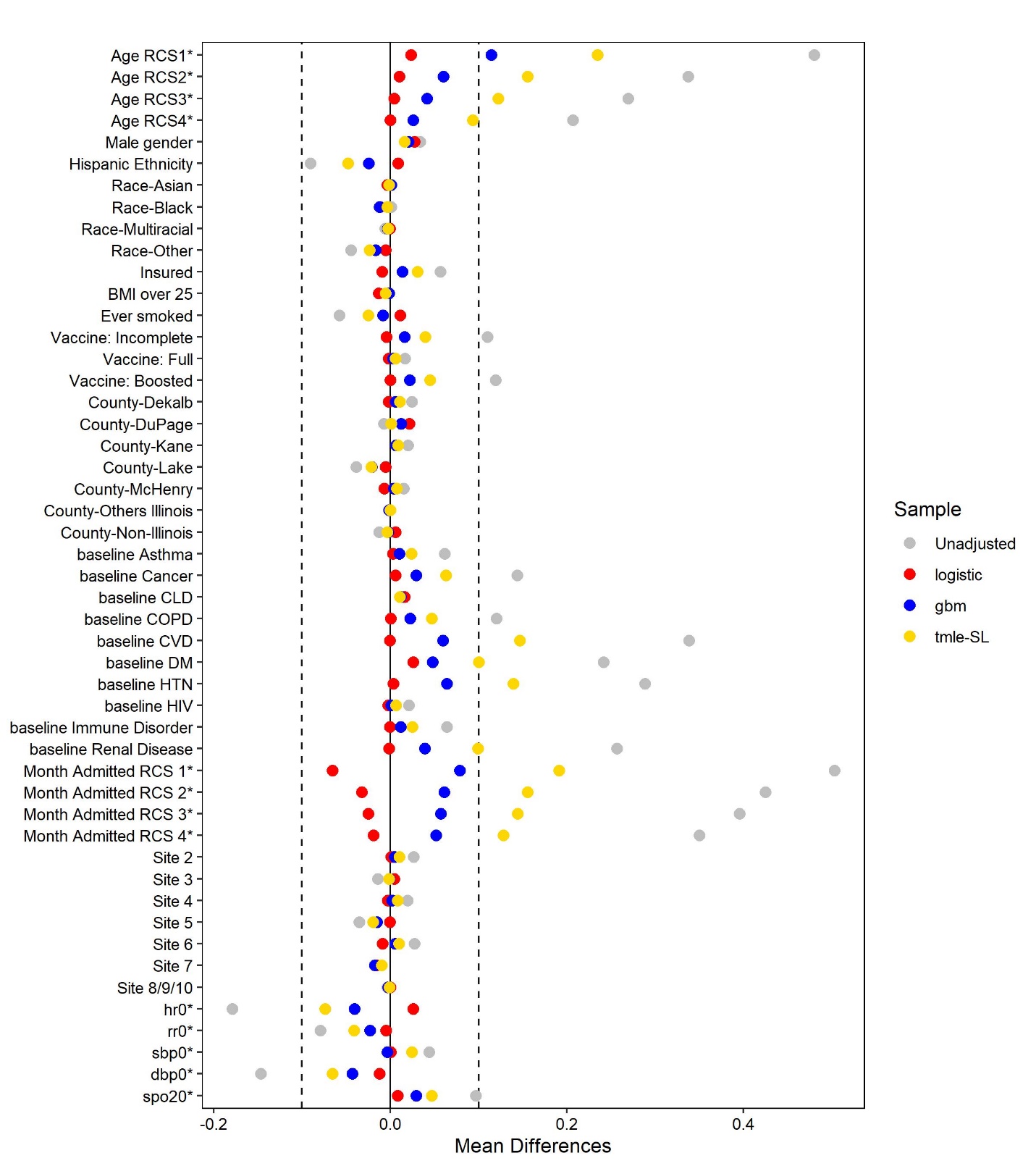


Note: * - standardized mean differences. Unadjusted – no weighting applied to calculated differences. logistic – used weights derived from a logistic regression model. gbm – weights derived from the a gradient boosted model. tmle-SL – weights obtained from the targeted maximum likelihood estimation object with super learner. BMI – body mass index, CLD – chronic liver disease, COPD – chronic obstructive pulmonary disease, CVD – cardiovascular disease, DBP0 – baseline diastolic blood pressure, DM – diabetes mellitus, HTN – hypertension, HIV – human immunodeficiency virus, HR0 – baseline heart rate, RCS – restricted cubic spline. RR0 – baseline respiratory rate, SBP0 – baseline systolic blood pressure, SPO20 – baseline oxygen saturation. Multiracial individuals are people who report more than two categories (e.g., Asian and Black), Other races include American Indian, Alaska Native, Native Hawaiian, Pacific-Islander, Guamanian, and Chamorro or chose other or none of the above. Other Illinois counties include all other counties not listed in the figure.
